# Spatial–temporal distribution patterns and influencing factors analysis of comorbidity prevalence of chronic diseases among middle-aged and elderly people in China: focusing on exposure to ambient fine particulate matter (PM_2.5_)

**DOI:** 10.1186/s12889-024-17986-0

**Published:** 2024-02-22

**Authors:** Liangwen Zhang, Linjiang Wei, Ya Fang

**Affiliations:** 1 State Key Laboratory of Molecular Vaccinology and Molecular Diagnostics, School of Public Health, Xiamen University, Xiamen, China; 2 Key Laboratory of Health Technology Assessment of Fujian Province, School of Public Health, Xiamen University, Xiamen, China

**Keywords:** Multimorbidity, GTWR, Spatial analysis, Air pollution, Cohort study

## Abstract

**Objective:**

This study describes regional differences and dynamic changes in the prevalence of comorbidities among middle-aged and elderly people with chronic diseases (PCMC) in China from 2011–2018, and explores distribution patterns and the relationship between PM_2.5_ and PCMC, aiming to provide data support for regional prevention and control measures for chronic disease comorbidities in China.

**Methods:**

This study utilized CHARLS follow-up data for ≥ 45-year-old individuals from 2011, 2013, 2015, and 2018 as research subjects. Missing values were filled using the random forest machine learning method. PCMC spatial clustering investigated using spatial autocorrelation methods. The relationship between macro factors and PCMC was examined using Geographically and Temporally Weighted Regression, Ordinary Linear Regression, and Geographically Weighted Regression.

**Results:**

PCMC in China showing a decreasing trend. Hotspots of PCMC appeared mainly in western and northern provinces, while cold spots were in southeastern coastal provinces. PM_2.5_ content was a risk factor for PCMC, the range of influence expanded from the southeastern coastal areas to inland areas, and the magnitude of influence decreased from the southeastern coastal areas to inland areas.

**Conclusion:**

PM_2.5_ content, as a risk factor, should be given special attention, taking into account regional factors. In the future, policy-makers should develop stricter air pollution control policies based on different regional economic, demographic, and geographic factors, while promoting public education, increasing public transportation, and urban green coverage.

**Supplementary Information:**

The online version contains supplementary material available at 10.1186/s12889-024-17986-0.

## Introduction

In the context of global population aging, the life expectancy of populations in various countries and regions has been increasing almost universally. However, with aging often comes the occurrence of chronic diseases [1], leading to a continuous increase in the number of patients with chronic non-communicable diseases and a growing prevalence of multimorbidity [2, 3]. China, as the most populous developing country with a large elderly population, has entered an era of deep aging. According to the 7th National Population Census, the proportion of people aged over 65 has reached 13.5% [4, 5]. With the increase in life expectancy and the widespread prevalence of various risk factors, the number of chronic disease patients in China continues to expand. Additionally, the situation of multiple chronic diseases coexisting is becoming increasingly serious [6]. Chronic diseases are the main source of global disease burden [7, 8] and have become a focus problem that seriously troubles individuals, families, and society. They are also the biggest obstacle to achieving the "Healthy China 2030" goal [9, 10]. In 2019, deaths caused by chronic diseases accounted for 88.5% of the total deaths in China [10]. Compared to having a single chronic disease, the threat of comorbidities of chronic diseases to patients' life safety and quality of life is greater, and the risk of death is higher [11]. Studies have shown that for each additional disease, the average life expectancy of patients will be shortened by 1.8 years [12–14]. Therefore, it is of great practical significance to clarify the influencing factors of the prevalence of comorbidities among middle-aged and elderly people with chronic diseases (PCMC). This will help improve the quality of life and happiness of middle-aged and elderly people and ensure that the elderly can enjoy their later years.

Environmental air pollution has become a global public health problem, and fine particulate matter ((PM_2.5_) is one of its main components. PM_2.5_ has a small diameter and a large surface area, which allows it to carry various toxic substances. It can enter the bloodstream directly through the blood-gas barrier, causing damage to various tissues and organs of the body and posing potential hazards to health. Previous studies have shown that PM_2.5_ can harm the physical function of middle-aged and elderly individuals [12–15]. However, it is still uncertain whether PM_2.5_ exposure is associated with the occurrence of chronic disease comorbidity. Further research is needed to investigate this relationship.

Researchers have gradually recognized that the etiology of chronic diseases is complex and involves not only genetics and unhealthy lifestyles but also spatial factors such as geographical location, environment, climate, and various levels of pollution from harmful substances [16, 17]. Therefore, it is necessary to analyze the distribution of various disease patterns by exploring their associations with environmental and geographical factors. Reviewing the studies conducted by scholars, it can be observed that most of the current research focuses on individual chronic diseases, such as diabetes and hypertension. Significant progress has been made in understanding their spatial distribution characteristics, providing data support for the development of regional control measures for chronic diseases [18–20]. In terms of studying multimorbidity, scholars have utilized methods like spatial autocorrelation or spatial clustering to identify the spatial distribution of comorbid chronic diseases. For instance, Guo Xiaorong et al. used spatial autocorrelation methods to investigate the spatial distribution and patterns of multimorbidity among the elderly in China in 2015 [21]. Isabel et al. employed Bernoulli cluster analysis to determine regional disparities in the burden of comorbid diseases [22]. Regarding the analysis of influencing factors, current studies often employ basic global or local regression methods to analyze cross-sectional data. For example, Peixi Rong et al. identified the influencing factors of multimorbidity among the elderly in China but did not consider environmental variables [23]. However, it is evident that the research on multimorbidity is still incomplete and has certain limitations. Many studies rely on cross-sectional data, neglecting the lag effects of various influencing factors and failing to capture the dynamic factors affecting multimorbidity in middle-aged and elderly individuals. Furthermore, there is limited exploration of the actual effects of air pollution, a significant influencing factor for comorbidities.

It is worth noting that spatial statistical methods have rapidly evolved in recent years, leading to the development of a series of more advanced and targeted local spatial analysis methods. These methods have been widely applied by scholars in various fields such as land allocation, environmental pollution, and urban planning [24, 25]. For example, Shukui Tan utilized multiscale geographically weighted regression (MGWR) to analyze the different socioeconomic driving factors influencing carbon emissions, identifying the scales of influence for each factor [26]. Maomao Zhang employed MGWR to differentiate the socioeconomic factors affecting land transfer scale [27]. Xiyu Zhang introduced temporal weighted regression (GTWR) to analyze the dynamic influencing factors of interprovincial occurrence rates of catastrophic health expenditures using longitudinal data [28]. The GTWR model is a spatial and temporal weighted regression method that introduces spatial and temporal weights to the traditional linear regression model. It allows the model parameters to vary in both spatial and temporal dimensions, thereby taking into account the non-stationarity in space and time. The GTWR model has enhanced predictive capability and higher interpretability compared to traditional models [29]. It is particularly suitable for utilizing longitudinal data to analyze the dynamic environmental influencing factors of chronic diseases.

**Fig. 1:**
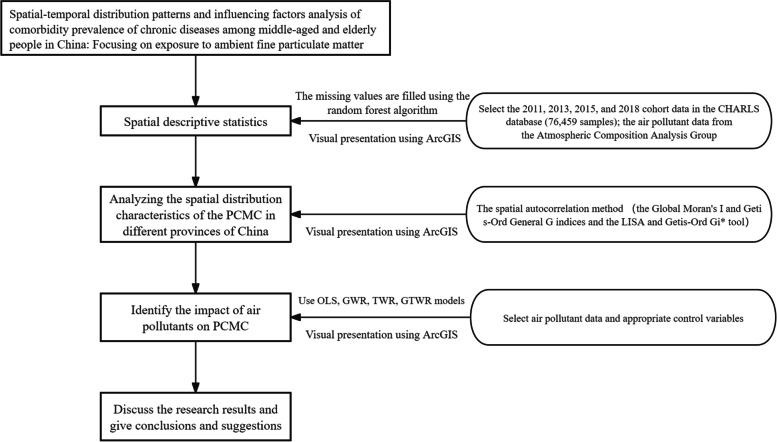
Research framework

Therefore, this study utilized national authoritative data and data from the China Health and Retirement Longitudinal Study (CHARLS) to analyze the spatial distribution characteristics of PCMC in different provinces of China. Firstly, the spatial autocorrelation method is used to analyze the spatial distribution characteristics of the PCMC in different provinces of China, and to identify aggregation and dispersion regions. Secondly, based on the GTWR model, which considers spatial and temporal heterogeneity, the relationship between the PCMC and macro factors such as environmental PM_2.5_ content is modeled. The coefficient function was estimated to reveal spatiotemporal heterogeneity, and the results were compared with those of the Ordinary Linearity Regression (OLR) and Geographically Weighted Regression (GWR) models. Finally, based on the estimation results of the coefficient function, the effects of different macro factors on different regions were investigated, and the research results were visualized using GIS technology (See Fig. 1 for details).

The aim of this study was to expand and deepen the theoretical research in the field of chronic disease comorbidity. Additionally, it aimed to provide a theoretical basis and decision-making support for the construction and optimization of regionalized prevention and control measures for chronic disease comorbidity in China. This study has two potential innovative aspects: (1) It utilizes longitudinal panel data to analyze the spatial distribution, regional disparities, and temporal trends of the co-occurrence of air pollution and chronic diseases.(2) Taking into account both spatial and temporal dimensions, it employs various regression methods (OLS, GWR, TWR, GTWR) to investigate the impact of air pollutants (PM_2.5_) on the comorbidity of chronic diseases among middle-aged and elderly populations. The study also considers the influence of regional heterogeneity and temporal heterogeneity, thereby capturing the lagged effects of air pollution on the comorbidity of chronic diseases to some extent.

## Methods

### Data acquisition

Based on the principle of data accessibility, this study selected all provincial-level units except for Hong Kong, Macau, Taiwan, Hainan, Ningxia, and Tibet as the study area. Data from four time points (2011, 2013, 2015, and 2018) were selected to evaluate the spatiotemporal distribution characteristics of the PCMC and to analyze the impact of fine particulate matter on comorbidities of chronic diseases and its spatiotemporal non-stationarity. The data on comorbidities of chronic diseases among middle-aged and elderly people were obtained from the China Health and Retirement Longitudinal Study (CHARLS), which used a multi-stage sampling method to cover 28 provincial-level units in China and has a certain national representativeness.

This study obtained high spatial resolution ground-level PM_2.5_ concentration data from the Atmospheric Composition Analysis Group at Dalhousie University [30, 31]. The data were based on the Twin MODerate Resolution Imaging Spectroradiometer (MODIS), Multiangle Imaging SpectroRadiometer (MISR), and Sea-viewing Wide Field-of-view Sensor (SeaWIFS) of the US National Aeronautics and Space Administration (NASA) inversed to obtain aerosol optical depth (AOD) data, combined with the GEOS-Chem chemical transport model, and ground monitoring data, which were incorporated into the GWR to obtain ground-level annual PM_2.5_ concentration in China with a resolution of 0.01°*0.01°.

Next, geocoding was performed on individual addresses, and the PM_2.5_ concentration measurements were assigned using ArcGIS software (ESRI Corporation). Specifically, the average concentration for each grid cell (0.01° × 0.01°) was merged with a geographic shapefile containing the boundaries of Chinese provinces. The average PM_2.5_ exposure concentration was then allocated to each province, resulting in annual provincial-level PM_2.5_ mean data for the years 2011, 2013, 2015, and 2018. The remaining environmental control variable data were sourced from the 2012, 2014, 2016, and 2019 editions of the "China Statistical Yearbook" and the "China Civil Administration Statistical Yearbook.".

### Sample selection

This study included the prevalence of 14 common chronic diseases in the health and function questionnaire of CHARLS, which were self-reported by the respondents. The chronic diseases included hypertension, dyslipidemia, diabetes or elevated blood glucose, cancer, chronic lung disease (such as chronic bronchitis or emphysema, pulmonary heart disease), liver disease, heart disease (such as myocardial infarction, coronary heart disease, angina pectoris, congestive heart failure, and other heart diseases), stroke, kidney disease, gastric disease, emotional and mental health problems, memory-related diseases (such as Alzheimer's disease, cerebral atrophy, Parkinson's disease), arthritis, and asthma. The definition of middle-aged and elderly people in this study was age ≥ 45 years [32, 33], and the definition of comorbidities of chronic diseases was the coexistence of two or more chronic diseases [34]. To address the issue of missing values in the study sample, this study utilized machine learning techniques and the random forest imputation method.

In the regression analysis, the PCMC in each province was taken as the dependent variable, and the impact of PM_2.5_ concentration was explored [35, 36]. Based on the research of other scholars and the underlying logic of changes in PCMC [37, 38], this study selected macro factors such as provincial area, population density, population dependency ratio, hospital quantity, basic medical institution quantity, and specialized public health institution quantity as control variables.

### Statistical methods

#### Spatial autocorrelation

Spatial Autocorrelation is commonly used to explore whether there is statistical correlation between a certain variable or data in space, or to explore the potential mutual influence among several data indicators. The research theory inherits the first law of geography proposed by Swiss geographer Tobler, which states that everything is related to everything else, but near things are more related [39]. As spatial autocorrelation can discover the distribution status and regular features of data in space, such as exploring the aggregation and dispersion states of data distribution, exploring the hot and cold spots of data distribution, it is often used to study the distribution of related indicators of public service facilities. In this study, the Global Moran's I and Getis-Ord General G indices and the local indicator of spatial autocorrelation (LISA) and Getis-Ord Gi* tool were used to analyze the spatial distribution characteristics of the PCMC in each province, and to identify the aggregation and dispersion regions of data distribution.

##### Global spatial autocorrelation

Global Moran's I reflect the overall spatial autocorrelation of the study area and is used to determine whether there is spatial autocorrelation in the research object as a whole. It is a spatial autocorrelation statistic for the entire study area [40]; The Getis-Ord General G method is used to preliminarily determine the clustering type.

The Global Moran’s I method of global spatial autocorrelation is given as

$$I = \frac{n}{{S}_{0}} \times \frac{{\sum }_{i=1}^{n}{\sum }_{j=1}^{n}{W}_{ij} \left({y}_{i} - \overline{y}\right) \left({y}_{j} - \overline{y}\right)}{{\sum }_{i=}^{n}{\left({y}_{i} - \overline{y}\right)}^{2}} , {S}_{0} \sum_{i=1}^{n}\sum_{j=1}^{n}{W}_{ij}$$

The Getis-Ord General G method of global spatial autocorrelation is given as

$${\text{G}} = \frac{{\sum }_{i=1}^{n}{\sum }_{j=1}^{n}{W}_{i,j} {x}_{i} {x}_{j}}{{\sum }_{i=1}^{n}{\sum }_{j=1}^{n}{x}_{i} {x}_{j}} , j \ne 1$$

The range of values for Global Moran's I is [-1, 1]. Observing Moran's I can help determine the aggregation and dispersion status of data in space. If Moran's I < 0, it indicates that the statistical data exhibit negative correlation in space, and the closer Moran's I is to -1, the stronger the negative correlation. If Moran's I = 0, it indicates that there is no obvious correlation in the spatial distribution of the statistical data, and the data are randomly and uniformly distributed. If Moran's I > 0, it indicates that the statistical data exhibit positive correlation in space, and the closer Moran's I is to 1, the stronger the positive correlation. In the analysis results of Getis-Ord General G, if the *p*-value is significant and the z-score is greater than 0, the higher the score, the tighter the clustering of high-value (hotspot) clusters; if the z-score is less than 0, the lower the score, the tighter the clustering of low-value (coldspot) clusters.

##### Local spatial autocorrelation

The global autocorrelation statistic indicates the presence of clustering, while the local autocorrelation indicates the location and type of spatial correlation. In order to further study the distribution pattern of the PCMC, the local autocorrelation analysis method was used to identify the reachable local clusters. Due to the heterogeneity of space, there will be different clustering states in different geographical locations. LISA is suitable for studying the heterogeneity characteristics of the aggregation of PCMC [41]. The Getis-Ord Gi* tool is applicable for hotspot analysis, which can analyze the distribution of cold spots and hotspots of the PCMC.

Its calculation expression is as follows:

The LISA method of local spatial autocorrelation is given as

$${I}_{i} = \frac{n\left({x}_{i} - \overline{x}\right) {\sum }_{i=1}^{n}{\sum }_{j=1}^{n}{w}_{i,j}\left({x}_{i} - \overline{x}\right)}{{\sum }_{i=1}^{n}{\left({x}_{i} - \overline{x}\right)}^{2}}$$

The Getis-Ord Gi* method of local spatial autocorrelation is given as

$${G}_{i}^{*} = \frac{{\sum }_{j=1}^{n}{W}_{i,j} {x}_{j} -\overline{X} {\sum }_{j=1}^{n}{w}_{i,j}}{{S}^{2}\sqrt{\left[{\frac{n{\sum }_{j=1}^{n}{w}_{i}^{2} - \left({\sum }_{j=1}^{n}{W}_{i,j}\right)}{n -1}}^{2}\right]}} ,$$

where and are attribute values for features i and j; is the spatial weight between feature i and feature j; and n is the number of features in the dataset. When the statistic is higher than the mathematical expectation and passes the hypothesis test, it is a hot spot; otherwise, it is a cold spot [42].

#### Regression model

The first step is to use the OLS regression model to fit the relationship between the PCMC and the annual average PM_2.5_ content in each province in China, and calculate the variance inflation factor (VIF) of each independent variable [43] to test for multicollinearity.

Secondly, in the selection of spatial regression methods, scholars in the research on spatial heterogeneity and spatial effects often use the GWR model as a local spatial regression model [44], which can determine the local influence factors of the PCMC in different spatial locations. However, GWR can only model cross-sectional data and does not consider changes over time, so the conclusions obtained are not complete. In order to accurately fit the time and space effects and make the regression model estimates more accurate, Huang Bo et al. proposed the GTWR model [45, 46], which introduces the time dimension, uses geographic location and time scale functions to calculate each local regression equation, and the regression parameters of the independent variables in the model vary with the change of spatiotemporal location, providing strong support for analyzing the spatiotemporal characteristics of regression relationships.

The general form of the two models is as follows:

1 $${{\text{y}}}_{{\text{i}}} = {\beta }_{0} \left({u}_{i},{v}_{i}\right) + \sum_{k=1}^{P}{\beta }_{k} \left({u}_{i},{v}_{i}\right) {x}_{ik} + {\varepsilon }_{i}$$

2 $${{\text{y}}}_{{\text{i}}} = {\beta }_{0} \left({u}_{i},{v}_{i},{t}_{i}\right) + \sum_{k=1}^{P}{\beta }_{k} \left({u}_{i},{v}_{i},{t}_{i}\right) {x}_{ik} + {\varepsilon }_{i}$$

Formula (1) represents the GWR model, Formula (2) represents the GTWR model, where ${y}_{i}$ represents the value of the dependent variable at study unit i; $\left({u}_{i},{v}_{i},{t}_{i}\right)$ represents the longitude, latitude, and time coordinates of the i-th sample point; ${\beta }_{0} \left({u}_{i}, {v}_{i}, {t}_{i}\right)$ is the regression intercept of study unit i, ${\beta }_{k} \left({u}_{i}, {v}_{i}, {t}_{i}\right)$ is the regression coefficient of the kth explanatory variable on study unit i, ${x}_{ik}$ is the data of the kth explanatory variable on study unit i, ${\varepsilon }_{i}$ is the Error term which meets the ${\varepsilon }_{i} \sim N \left(0, {\sigma }^{2}\right)$ assumption.

The choice of bandwidth can affect the results of the model. A bandwidth that is too small may lead to overfitting, while a bandwidth that is too large may include points that have little effect on the model, leading to inaccurate results. In this study, an adaptive bandwidth selection method was used, which selects the bandwidth and model based on the modified Akaike Information Criterion (AICc) [47]. Furthermore, to demonstrate the superior fitting performance of the GTWR model, this study compared the results of the OLS regression model, traditional GWR model, and TWR model. OLS was chosen as the comparison method due to its wide applicability, global nature, and unbiasedness [48].

In addition, this study used SPSS 26.0 software for data management and OLS analysis, and ArcGIS 10.8 software with the GTWR plugin [45] for spatial autocorrelation and spatial regression analysis. A two-tailed test with a significance level of α = 0.05 was used for hypothesis testing.

#### Model evaluation

To evaluate the model fit and complexity, this study utilized metrics such as the Coefficient of Determination (R2), Adjusted R-squared (AdjR2), Akaike Information Criterion with correction (AICc), and Residual Sum of Squares (RSS). These metrics were used to compare different models and select the optimal regression model. R2 and AdjR2 were used to assess the goodness of fit of the model, with higher values indicating better fit. AICc was used to compare the complexity of the models, with lower values indicating simpler models. RSS was used to evaluate the magnitude of the residuals, with lower values indicating better model performance.

## Results

### Description

This study included a total of 76,459 samples. The sample size for each year was 17,502 cases in 2011, 18,420 cases in 2013, 20,838 cases in 2015, and 19,684 cases in 2018. In 2011, among middle-aged and elderly people, there were 5,735 cases (32.8%) without chronic diseases, 5,133 cases (29.3%) with one chronic disease, and 6,634 cases (37.9%) with two or more chronic diseases. In 2013, the corresponding numbers were 7,483 cases (40.6%), 4,523 cases (24.6%), and 6,414 cases (34.8%). In 2015, there were 8,851 cases (42.5%), 4,777 cases (22.9%), and 7,210 cases (34.6%), while in 2018, there were 10,832 cases (55.0%), 4,337 cases (22.0%), and 4,515 cases (22.9%). The prevalence of various chronic diseases is shown in Table S1.

**Fig. 2:**
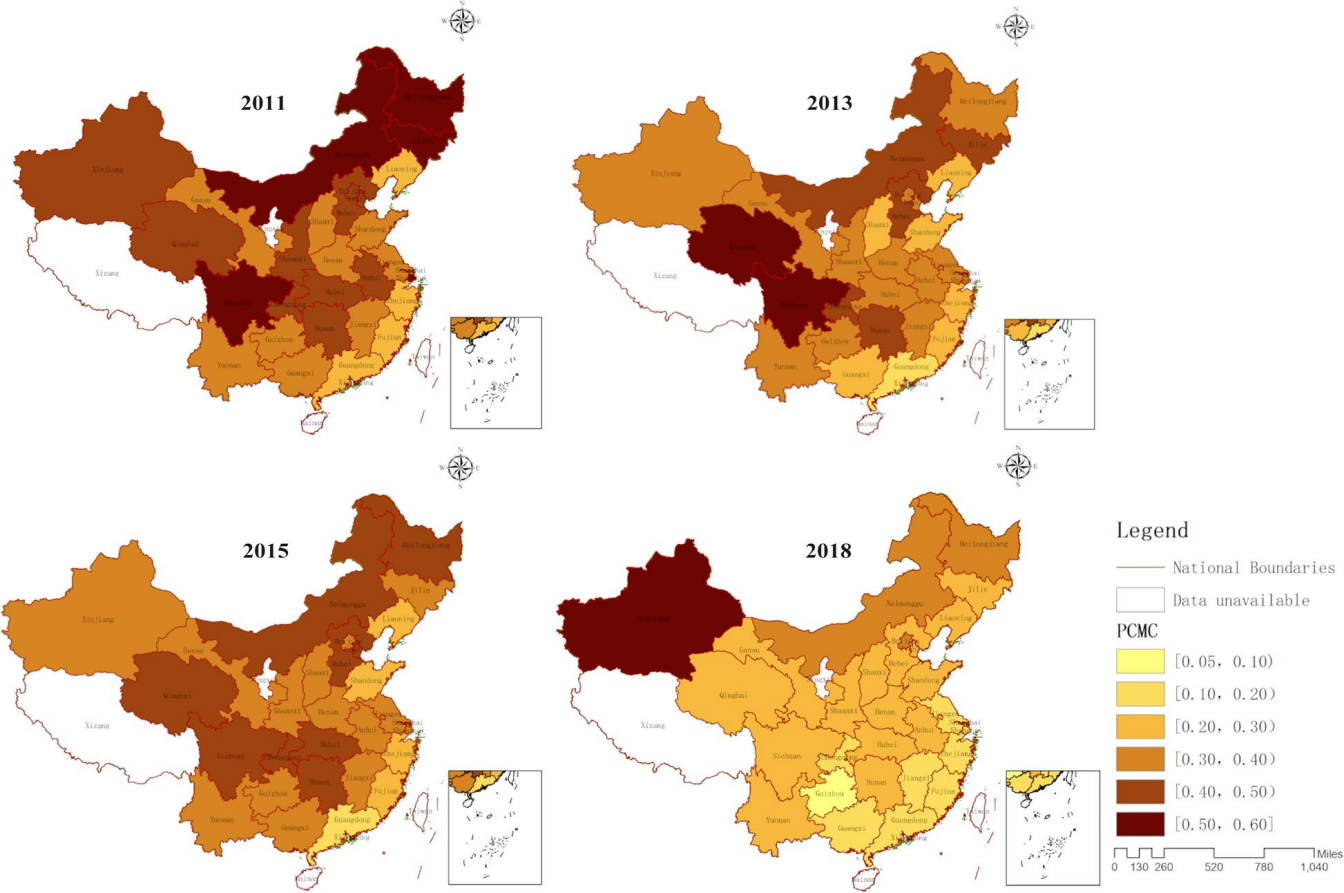
Dynamic changes in PCMC across provinces

Figure 2 shows the PCMC in different provinces of China. The PCMC in China has shown a decreasing trend over time. In 2011, Guangdong province had the lowest PCMC, which was 20.95%. Four provinces had a PCMC over 50%, namely Jilin province, Heilongjiang province, Inner Mongolia, and Sichuan province. By 2018, Guizhou province had the lowest PCMC, which was only 9.05%. Xinjiang was the only province with a PCMC over 50%, while the prevalence in the rest of the provinces was below 40%. In terms of regional distribution, provinces located in the southeastern coastal areas consistently had lower PCMC. The PCMC in northeastern provinces has been well controlled, while there is still room for improvement in western regions.

**Fig. 3:**
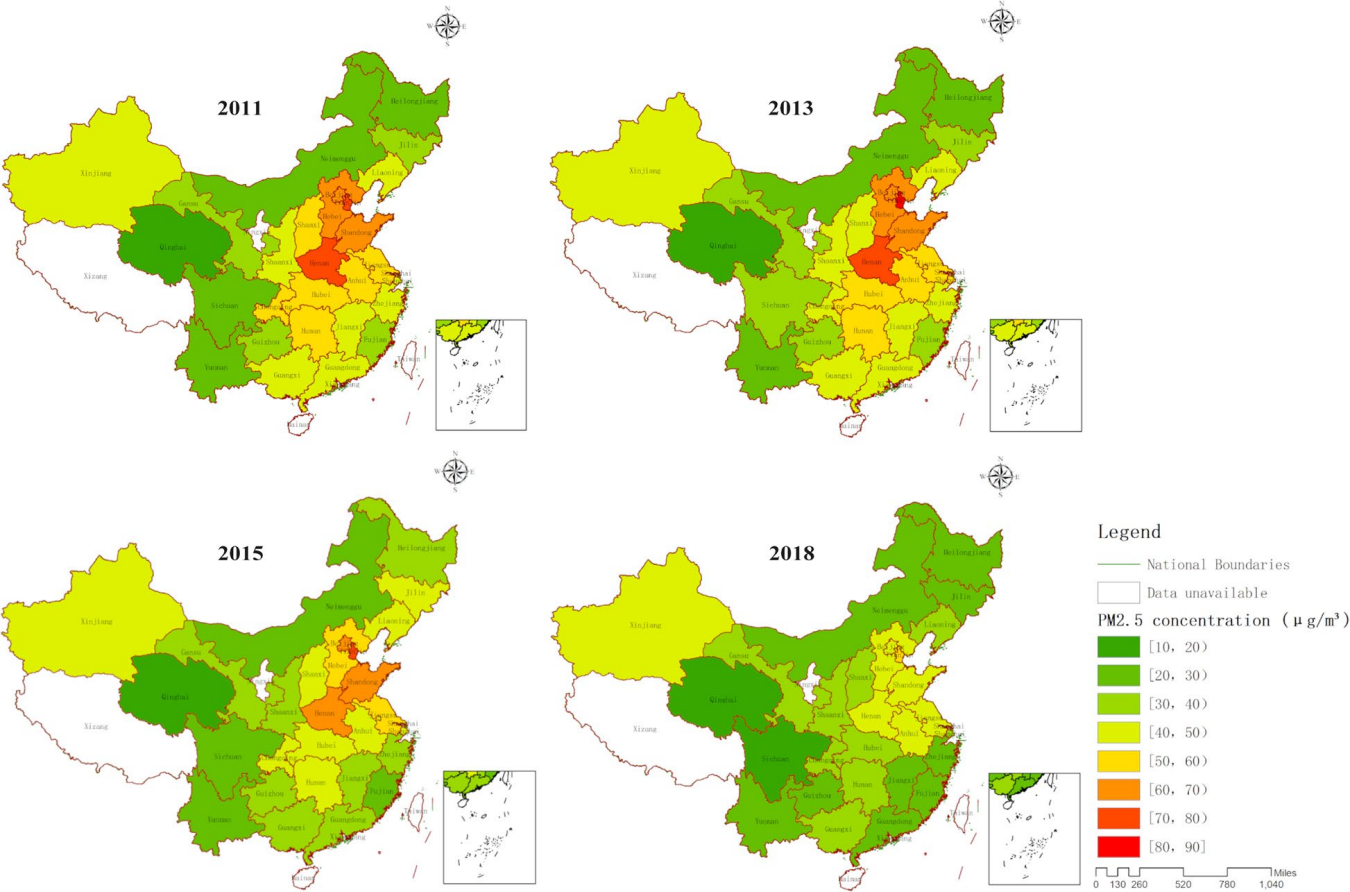
Dynamic changes in annual average PM2.5 content across provinces

Figure 3 shows that the level of PM_2.5_ pollution in China has also shown a decreasing trend since 2013. Tianjin has consistently had the highest annual average PM_2.5_ content for four years. In 2011, its value was 79.39*μg/m*^*3*^, which increased to 82.49*μg/m*^*3*^ in 2013, but then began to decrease. By 2015, it had decreased to 73.06*μg/m*^*3*^, and in 2018, it was only 52.73*μg/m*^*3*^. Qinghai has consistently had the lowest annual average PM_2.5_ content among all provinces, with a trend similar to that of Tianjin. In 2011, its value was 12.84*μg/m*^*3*^, which increased to 13.70*μg/m*^*3*^ in 2013, decreased to 12.39*μg/m*^*3*^ in 2015, and continued to decrease to 12.19*μg/m*^*3*^ in 2018. Provinces with severe PM_2.5_ pollution are mainly located in developed regions in the east, with Beijing and its surrounding provinces experiencing particularly severe pollution. In addition, Xinjiang is the only province where the PM_2.5_ content has not decreased in recent years. In 2011, its value was 44.96*μg/m*^*3*^, and in 2018, it was 46.21*μg/m*^*3*^.

### Spatial statistical analysis of PCMC

#### Spatial autocorrelation analysis of PCMC

The results of global spatial autocorrelation for the PCMC in each province are presented in Table 1 and Figures S7 and S8. The Global Moran's I results show that there was no spatial autocorrelation in the PCMC in 2011 and 2013. However, in 2015, positive spatial autocorrelation began to emerge within the 90% confidence interval. By 2018, there was strong positive spatial autocorrelation within the 99% confidence interval. This indicates that the spatial distribution of the PCMC has gradually shown positive correlation over time, with clustering in areas of high prevalence and low prevalence. This study further conducted Getis-Ord General G calculation, but the results for four years did not show significance, indicating that there may not be significant high-value or low-value clustering areas nationally. Therefore, further local spatial autocorrelation analysis is needed.

**Table 1:** Global autocorrelation analysis results

Year	Moran's I	z-score	*p*-value		observed General G	z-score	*p*-value
2011	0.017589	0.672866	0.501033		0.000001	-0.742472	0.457801
2013	0.034806	0.895831	0.370343		0.000001	-0.485638	0.627224
**2015**	**0.114417**	**1.868788**	0.061652*		0.000001	0.058729	0.953168
**2018**	**0.162901**	**2.597737**	0.009384*		0.000001	-0.176488	0.859911

^*^ The *P* value is statistically significant at the test level of 0.1

**Fig. 4:**
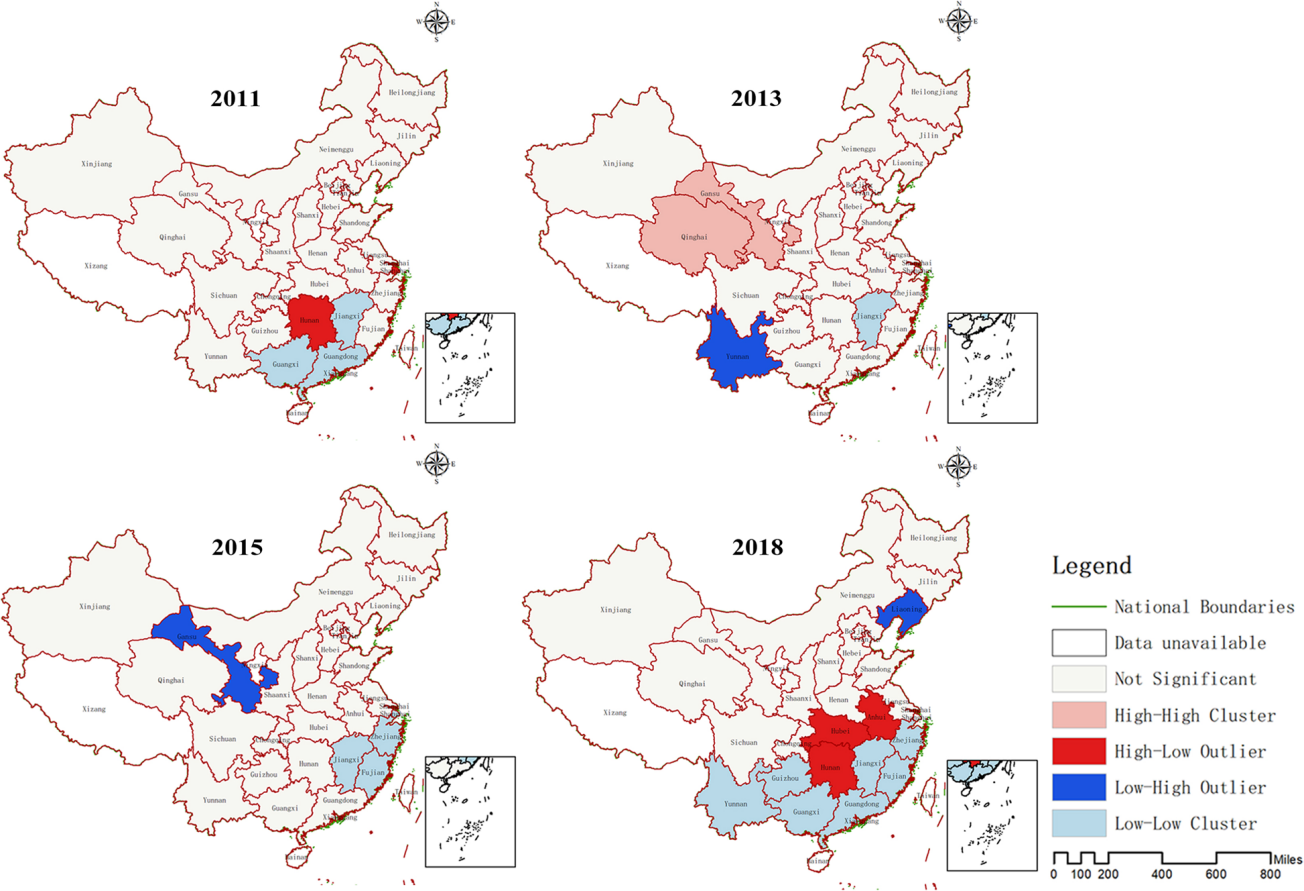
Clustering and outlier analysis results of PCMC

The LISA analysis results indicate that there is spatial heterogeneity in the PCMC in China. In 2011, Hunan province was classified as high-low, indicating that the PCMC in Hunan province was significantly higher than that in surrounding provinces. In 2013, Yunnan province was classified as low–high, indicating that the PCMC in Yunnan province was significantly lower than that in surrounding provinces. In 2015, Gansu province was classified as low–high, and in 2018, Liaoning province was classified as low–high, while Anhui, Hubei, and Hunan provinces were classified as high-low. Please refer to Fig. 4 for details.

**Fig. 5:**
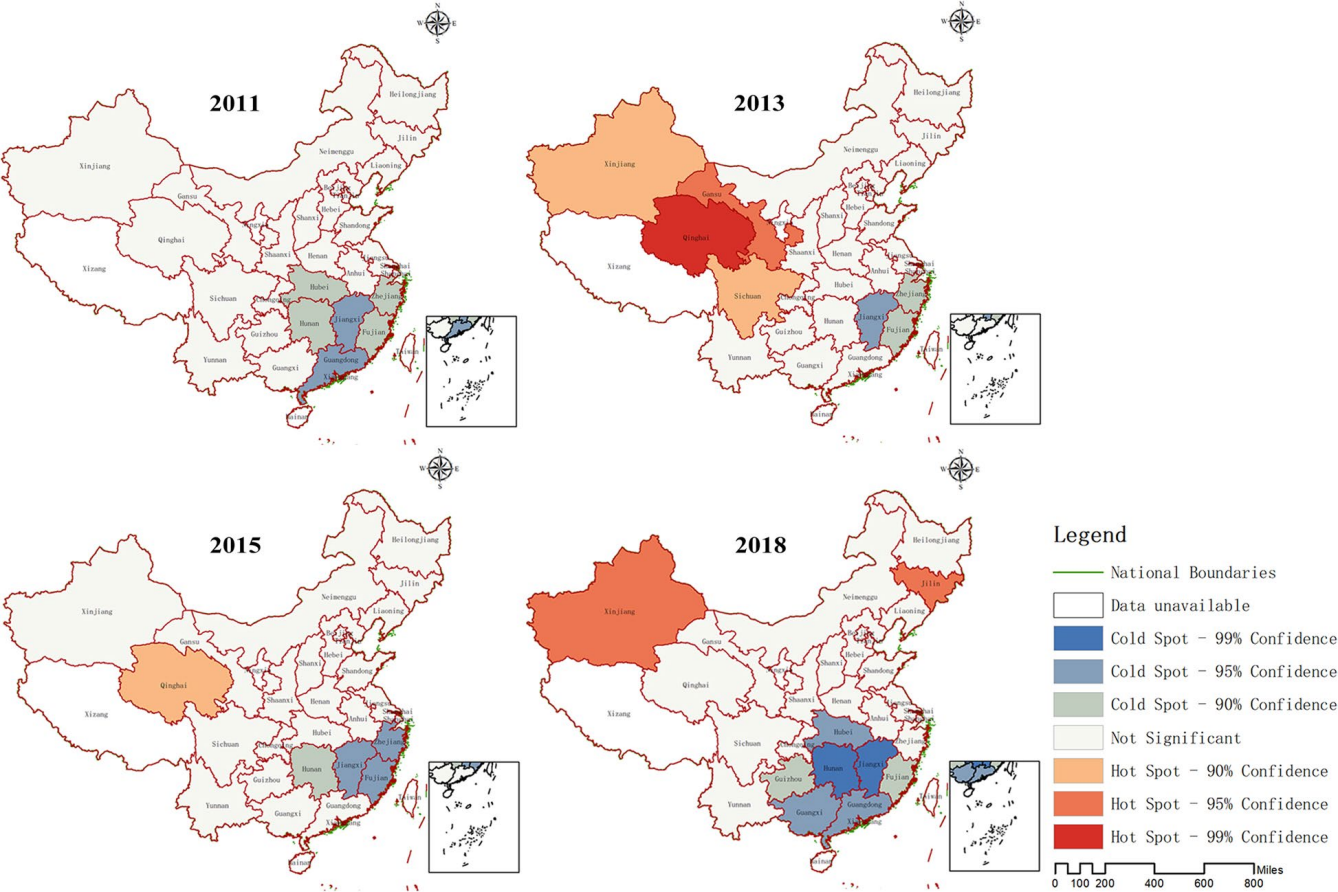
Analysis results of cold and hot spots of PCMC

The results of hot and cold spot analysis indicate that the cold spot areas of the PCMC in China from 2011 to 2018 were all located in southeastern provinces, and the range of these cold spots had expanded over time. By 2018, the cold spots had spread to seven provinces. In contrast, the hot spot areas were mainly located in western provinces of China, with Jilin province also becoming one of the hot spots by 2018. Please refer to Fig. 5 for details.

#### Regression analysis of PCMC

The results of the collinearity test presented in Table 2 show that the Tolerance values of all control variables are greater than 0.3, and the VIF values are all less than 5, which indicates that there is no collinearity between the control variables (The specific values and spatial distribution diagrams of the control variables included in the model are detailed in Table S1 and Figures S1-S6).

**Table 2:** Collinearity test results

	Unstandardized coefficient		Standardization coefficient	Covariance statistics	
	B	Standard error	Beta	tolerances	VIF
(Constant)	0.364	0.060			
Area	1.304E-07	0.000	0.449	0.772	1.295
Population density	-3.218E-06	0.000	-0.022	0.654	1.528
Population dependency ratio	-0.003	0.001	-0.200	0.727	1.376
Number of Hospitals	0.000	0.000	-0.501	0.400	2.502
Number of primary care institutions	1.652E-06	0.000	0.330	0.303	3.304
Number of professional public health institutions	1.314E-05	0.000	0.083	0.674	1.483
PM_2.5_ content	0.002	0.001	0.258	0.764	1.310

The overall fit results of different models are presented in Table 3. It can be observed that the GTWR model has the highest R2, the lowest AICc and RSS, and the AICc difference with other models exceeds 3, indicating a better fit [49]. Therefore, it can be concluded that the GTWR model provides a more accurate fit for the relationship between the PCMC and various influencing factors. In this study, the GTWR model was selected for further analysis, and an adaptive method was used to determine its bandwidth based on the sparsity of sample points to achieve a better fit.

**Table 3:** Model evaluation results

Evaluation Indicators	GTWR	OLS	GWR	TWR
R^2^	0.686191	0.441071	0.50651	0.509307
ADjustR^2^	0.66507	0.665432	0.473294	0.47628
AICc	-242.654	-238.249	-231.232	-229.648
RSS	0.376967	0.665432	0.592812	0.589452

**Fig. 6:**
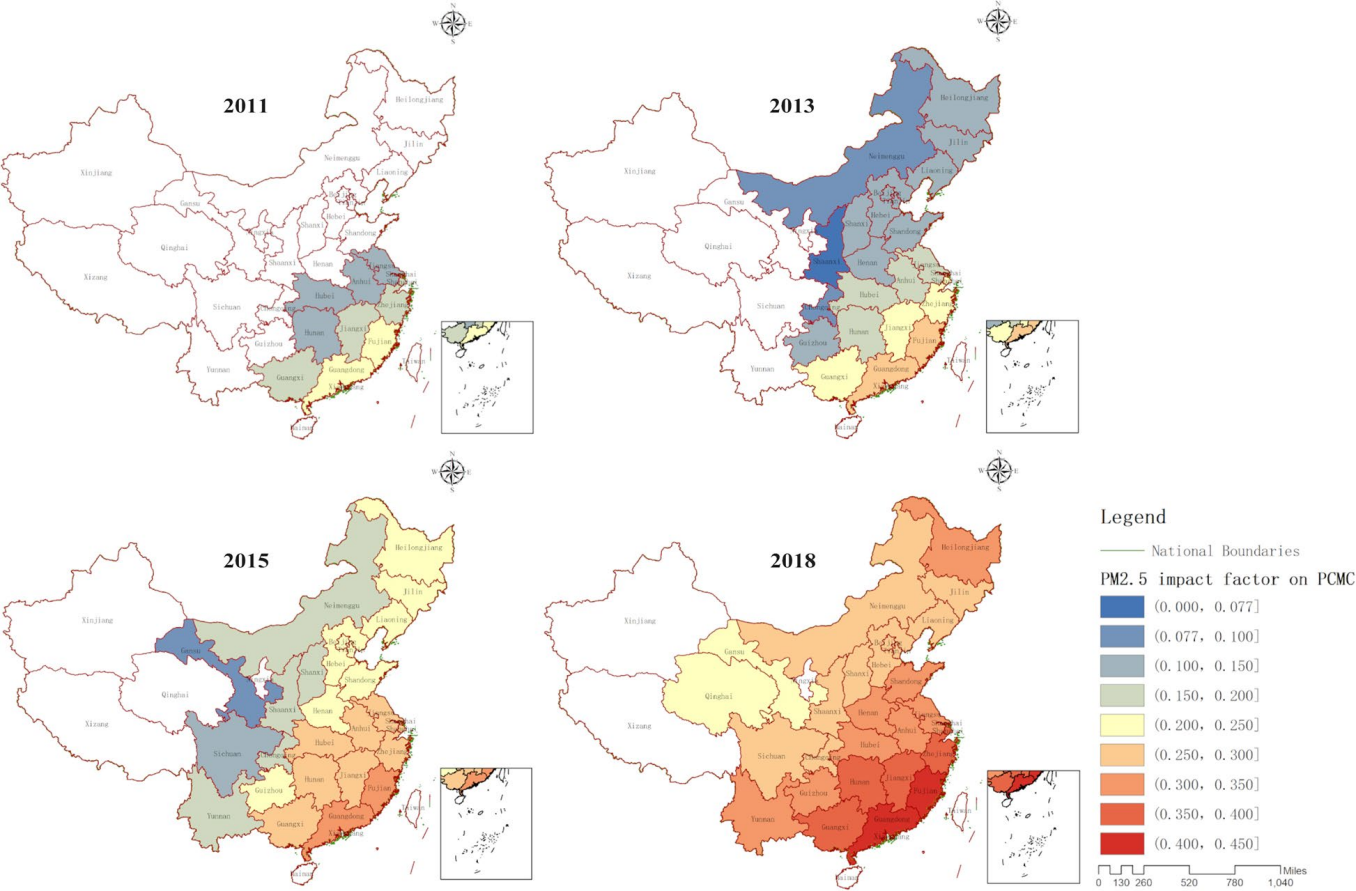
The Influence of Annual Average PM2.5 Content on PCMC in Various Years

The results of the GTWR model show that in 2011, only the PM_2.5_ content in the nine southeastern coastal provinces had a significant impact on the PCMC. However, over time, the PM_2.5_ content in more and more provinces showed a significant impact on PCMC, as shown in Fig. 6. The non-white provinces in Fig. 6 are the ones where the PM_2.5_ content has a significant impact on the PCMC. The color from blue to red indicates the increasing degree of impact, and the overall trend is that the impact range gradually expands from the southeastern coastal areas to the inland areas, and the impact size gradually decreases from the southeastern coastal areas to the inland areas. In 2011, Fujian province was the most affected, with a regression coefficient of 0.22, while Hubei province was the least affected, with a regression coefficient of 0.12. By 2018, Guangdong province was the most affected, with a regression coefficient of 0.41, while Qinghai province was the least affected, with a regression coefficient of 0.21, as shown in Table S3.

## Discussion

Since 2011, the implementation of the Healthy China strategy and the vigorous promotion of ecological civilization construction by the Chinese government, as well as the introduction of multiple related policies, have greatly alleviated the problem of chronic disease comorbidity and air pollution among middle-aged and elderly individuals in China. The PCMC and PM_2.5_ levels have both shown a declining trend. However, it is important to note that chronic disease comorbidity still poses a significant threat to the health of middle-aged and elderly individuals in China, and should be given special attention while considering regional factors. Continued efforts are needed to improve air quality, promote healthy lifestyles and behaviors, and ensure access to quality healthcare services for this population.

Overall, the PMAC in China has exhibited a declining trend over time, which can be attributed to the efforts made by the Chinese government in addressing and managing comorbid chronic diseases. The development of general practitioner teams has played a significant role in this regard [50], and the prioritization of advancing and enhancing the general practitioner system in China has helped to manage comorbidity effectively. Family doctor contract services have been implemented in major cities like Beijing, Shanghai, and Shenzhen, which are considered to be crucial in managing comorbidity [51]. However, in 2018, the PCMC in Xinjiang was still higher than 50%, which may be due to the vast geographical area, the low level of economic development, and the low accessibility of medical and elderly care resources. Additionally, a scarcity of non-medical health resources may also contribute to the high PCMC in Xinjiang. Continued efforts are needed to improve the accessibility of healthcare resources and non-medical health resources in underdeveloped regions to further reduce the PCMC in China [52].

Furthermore, China has indeed made remarkable achievements in controlling air pollution, with the annual average PM_2.5_ pollution level showing a downward trend since 2013. This can be attributed to the Chinese government's high attention to air pollution control, as evidenced by the release of the "Notice of the State Council on Printing and Distributing the Action Plan for Prevention and Control of Air Pollution" in 2013 [53], which put forward comprehensive governance requirements for industrial enterprise atmospheric pollution, surface source pollution, mobile source pollution, and other directions. During the period from 2011 to 2018, Tianjin consistently had the highest annual average PM_2.5_ content, with a value of 79.39*μg/m*^*3*^ in 2011, increasing to 82.49*μg/m*^*3*^ in 2013, but then showing a decreasing trend, dropping to 73.06*μg/m*^*3*^ in 2015, and reaching only 52.73*μg/m*^*3*^ in 2018.

This indicates that the efforts made by the Chinese government in controlling air pollution have been successful, and there is a positive trend in improving air quality in China. However, continued efforts are needed to further reduce the PM_2.5_ pollution level and improve air quality, especially in heavily polluted regions.

Based on the spatial autocorrelation analysis results, the spatial distribution of the PCMC has gradually exhibited positive correlation characteristics over time, showing spatial clustering, i.e., areas with high PCMC are surrounded by areas with high PCMC, and vice versa. However, there is spatial heterogeneity on a local level, with clear cold and hot spot areas. The main cold spot areas are in the southeastern coastal areas, while the main hot spot areas are in the western provinces of China. This may be determined by various factors such as regional environment, dietary habits, and economic development level. For example, the medical level in the southeastern coastal areas is relatively high, and people's health awareness is also relatively strong [54]. They attach more importance to the prevention and treatment of chronic diseases, which leads to a lower disease rate. In contrast, the medical conditions in the northwestern and inland provinces are relatively poor, and people's health awareness is relatively weak. They are more likely to neglect the prevention and treatment of chronic diseases, leading to a higher disease rate. Moreover, people in the southeastern coastal areas mainly eat seafood and vegetables, which are rich in nutrients such as dietary fiber, vitamins, and minerals [55]. These foods help reduce the incidence of cardiovascular and digestive system diseases. In contrast, people in the western provinces mainly eat meat and dairy products, which are high in fat and cholesterol, leading to a higher incidence of chronic diseases. Overall, these regional factors contribute to the spatial heterogeneity of the PCMC in China, highlighting the importance of targeted interventions and policies to address the different health needs of different regions.

The results of the regression analysis indicate that PM_2.5_ concentration is a risk factor contributing to the increased comorbidity of PCMC. Its impact has been expanding from the southeastern coastal regions towards inland areas over the years. This may be due to the duration of exposure to air pollutants, which is an important factor influencing the occurrence of chronic diseases among this age group. This finding has been widely supported by scholars [56–59]. Weuve et al. utilized medical statistical methods and found that higher levels of long-term exposure to PM_2.5_ were associated with a faster decline in cognitive abilities among older women [60]. Zhang et al., using data from the China Family Panel Studies, found significant impairments in cognitive performance due to air pollution with a lag of three years. Wang Y and Luo N discovered that the negative impact of air pollution on mental health mainly occurred within 0–9 months of pollution exposure, while the effects on physical health were concentrated within 9–18 months. However, their study did not explore long-term effects beyond 18 months due to data limitations [61]. In this study, we observe that over time, the impact of PM_2.5_ on PCMC becomes significant in an increasing number of provinces, which may further complement the limitations of Wang Y and Luo N's research. Additionally, we can observe that the magnitude of the impact of PM_2.5_ concentration on PCMC gradually decreases from the southeastern coastal regions to inland areas. This phenomenon may be influenced by factors such as the economic development level and natural environmental characteristics of each province [62].

The specific reasons may be as follows. The southeastern coastal areas are one of the most rapidly developing economic regions in China [63], with a fast urbanization process and relatively concentrated sources of pollution such as industry and transportation [64]. Therefore, the air quality has been affected earlier and more severely in this region [65]. In contrast, the inland regions have relatively backward economic development and fewer sources of pollution, resulting in the residents in these areas being affected by PM_2.5_ pollution later. The population density in the southeastern coastal areas is also relatively high, with a large concentration of people and human and production activities producing more pollutants, resulting in poor air quality. In contrast, the population density in the inland regions is relatively low, with fewer pollutants being emitted, resulting in a later impact on air quality. The urban construction in the southeastern coastal areas is relatively dense, with a significant urban heat island effect that affects air circulation and reduces air quality. In contrast, the urban construction in the inland regions is relatively dispersed, with a weaker urban heat island effect and relatively better air quality. Finally, the climate in the southeastern coastal areas is characterized by high temperature and short sunshine time, which may have a negative impact on air quality [66]. Inland regions, on the other hand, have lower temperatures and longer sunshine time, which are conducive to maintaining air quality [67].

Overall, these factors contribute to the regional differences in the impact of PM_2.5_ concentration on the PCMC in China, highlighting the importance of targeted interventions and policies to address the different health needs of different regions.

## Limitations

Although we attempted in this study to use various tests and methods to explore the relationship between annual average PM_2.5_ concentration and the PCMC in each province, there are still some limitations. Firstly, this study lacks strong causal inference evidence to prove the impact of annual average PM_2.5_ concentration on the PCMC, as there is currently limited research on the spatial non-stationarity of PCMC. However, this also implies that the research direction of this paper is innovative. Secondly, the CHARLS dataset is not sufficiently representative in terms of region, and the chronic disease data is collected from self-reported information from participants. To address and expand on these limitations, this study plans to further improve the quality of data, conduct field research, establish databases with regional representation, and expand the sample size in the future to correct these issues and expand on the results of this study.

## Conclusions and recommendations

This study provides valuable insights into the spatiotemporal patterns and factors affecting the PCMC in China from 2011 to 2018. The use of longitudinal data from a national perspective allows for a comprehensive analysis of the regional differences and dynamic changes in the PCMC, contributing to the development of the theoretical research in the field of chronic disease comorbidity. The study found that the PCMC in China has been greatly alleviated since 2011, showing a downward trend, which can be attributed to the high attention and strong promotion by the Chinese government. The spatial autocorrelation analysis showed that the PCMC had spatial clustering characteristics, with hotspots appearing in western or northern provinces and cold spots appearing in southeastern coastal provinces. The GTWR model results showed that the impact of PM_2.5_ concentration on the PCMC expanded gradually from southeast coastal areas to the inland regions, with the magnitude decreasing progressively from the southeast coastal areas to the inland regions.

These findings provide a good theoretical basis and decision-making reference for the construction and optimization of regional prevention and control measures for chronic disease comorbidity in China. By identifying the regional differences and factors affecting the PCMC, targeted interventions and policies can be developed to address the different health needs of different regions, contributing to the improvement of public health in China.

In conclusion, PM_2.5_ should be given priority attention as a health risk factor, considering regional factors. To construct a regional prevention and control system for chronic disease comorbidity and optimize the allocation of medical and health resources, this study proposes the following suggestions: Firstly, stricter policies for air pollution control should be formulated, and the supervision of polluting enterprises should be increased. Stricter emission standards should be adopted to reduce PM_2.5_ emissions [49]. Secondly, public transportation construction should be strengthened, and residents should be encouraged to reduce the use of private vehicles to reduce the impact of motor vehicle exhaust emissions on air quality [68]. Thirdly, publicity and education should be strengthened to increase public awareness of air pollution and chronic diseases. Residents should be encouraged to adopt positive health behaviors and lifestyles, such as exercising more, maintaining a healthy diet, quitting smoking, and limiting alcohol consumption [69]. Fourthly, the government should increase urban green coverage, introduce more plants and trees, enhance the ecological environment of cities, absorb harmful substances such as PM_2.5_, and improve urban air quality. Studies have shown that this measure can significantly improve air quality [70]. Finally, scientific research efforts should be strengthened, and research on air pollution and chronic diseases should be promoted. More scientific basis and support should be provided for the prevention and treatment of chronic diseases, and the treatment and prevention of chronic disease patients should be strengthened to improve the level and quality of medical services, thereby reducing the mortality and disability rates of chronic diseases [71].

### Supplementary Information

**Additional file 1:** **Table S1.** Prevalence of various chronic diseases. **Table S2.** Description of variables included in the regression model. **Table S3.** The coefficient and p-value of the impact of average PM2.5 content on PCMC in the GTWR. **Figure S1.** Spatial representation of the area of each province. **Figure S2.** Spatial representation of population density in each province. **Figure S3.** Spatial representation of population dependency ratio in each province. **Figure S4.** Spatial representation of hospital quantity in each province. **Figure S5.** Spatial representation of basic medical institution quantity in each province. **Figure S6.** Spatial representation of specialized public health institution quantity in each province. **Figure S7.** Global Moran's I result for PCMC in different years. **Figure S8.** Getis-Ord General G result for PCMC in different years.

## Data Availability

The data for this study were obtained from China Health and Retirement Longitudinal Research Study and publicly available government statistical yearbooks. Please contact the authors for data request.

## Abbreviation

PCMC: Prevalence of comorbidities among middle-aged and elderly people with chronic diseases
