# Supplementary material for: Spatial–temporal distribution patterns and influencing factors analysis of comorbidity prevalence of chronic diseases among middle-aged and elderly people in China: focusing on exposure to ambient fine particulate matter (PM2.5)
Source: BMC Public Health. 2024 Feb 22;24:550. doi: 10.1186/s12889-024-17986-0 (PMC10882846; doi:10.1186/s12889-024-17986-0)
Supplement: Supplementary file 1 — Additional file 1: Table S1. Prevalence of various chronic diseases. Table S2. Description of variables included in the regression model. Table S3. The coefficient and p-value of the impact of average PM2.5 content on PCMC in the GTWR. Figure S1. Spatial representation of the area of each province. Figure S2. Spatial representation of population density in each province. Figure S3. Spatial representation of population dependency ratio in each province. Figure S4. Spatial representation of hospital quantity in each province. Figure S5. Spatial representation of basic medical institution quantity in each province. Figure S6. Spatial representation of specialized public health institution quantity in each province. Figure S7. Global Moran's I result for PCMC in different years. Figure S8. Getis-Ord General G result for PCMC in different years. [file 12889_2024_17986_MOESM1_ESM.docx]

**Supplementary Information**

Additional file 1 (.docx) provides statistical descriptions and specific results of the equity, efficiency, and coordination analyses, as well as other additional information.

Table S1 Prevalence of various chronic diseases;

Table S2 Description of variables included in the regression model;

Table S3 The coefficient and p-value of the impact of average PM2.5 content on PCMC in the GTWR;

Figure S1 Spatial representation of the area of each province;

Figure S2 Spatial representation of population density in each province;

Figure S3 Spatial representation of population dependency ratio in each province

Figure S4 Spatial representation of hospital quantity in each province

Figure S5 Spatial representation of basic medical institution quantity in each province

Figure S6 Spatial representation of specialized public health institution quantity in each province

Figure S7 Global Moran's I result for PCMC in different years

Figure S8 Getis-Ord General G result for PCMC in different years

**Table S1** Prevalence of various chronic diseases

| Chronic disease | 2011(%) | 2013(%) | 2015(%) | 2018(%) |
| --- | --- | --- | --- | --- |
| Hypertension | 24.6 | 21.0 | 20.1 | 16.7 |
| Dyslipidemia | 9.5 | 8.3 | 8.5 | 12.7 |
| Diabetes or elevated blood glucose | 5.8 | 4.9 | 5.0 | 6.9 |
| Cancer | 1.0 | 0.8 | 1.0 | 1.3 |
| Chronic lung disease | 10.2 | 11.2 | 9.3 | 6.1 |
| Liver disease | 3.9 | 4.2 | 3.8 | 3.5 |
| Heart disease | 12.1 | 11.3 | 10.3 | 9.2 |
| Stroke | 2.4 | 1.8 | 2.1 | 5.4 |
| Kidney disease | 6.5 | 7.7 | 5.9 | 4.3 |
| Stomach problem | 22.4 | 22.5 | 21.5 | 9.0 |
| Emotional and psychiatric problem | 1.4 | 1.2 | 1.3 | 1.2 |
| Memory-related disease | 1.6 | 1.2 | 1.4 | 2.4 |
| Arthritis or rheumatism | 33.0 | 31.4 | 31.2 | 8.8 |
| Asthma | 3.7 | 3.5 | 3.5 | 2.4 |

**Table S2** Description of variables included in the regression model

| Year | Province | Provincial area (km²) | Population density (persons per km²) | Population dependency ratio | Hospital quantity | Basic medical institution quantity | Specialized public health institution quantity | Annual pm2.5 exposure concentration (μg/m3) | PCMC |
| --- | --- | --- | --- | --- | --- | --- | --- | --- | --- |
| 2011 | Anhui | 139700 | 426.39 | 38.89 | 916 | 21434 | 443 | 53.94 | 0.41 |
| 2011 | Beijing | 16800 | 1167.80 | 20.94 | 550 | 8718 | 121 | 69.52 | 0.41 |
| 2011 | Chongqing | 82300 | 350.50 | 40.29 | 433 | 17037 | 160 | 53.32 | 0.43 |
| 2011 | Fujian | 121300 | 304.45 | 30.48 | 482 | 26287 | 304 | 32.26 | 0.27 |
| 2011 | Gansu | 454400 | 56.34 | 35.85 | 385 | 25884 | 329 | 35.49 | 0.38 |
| 2011 | Guangdong | 180000 | 580.05 | 31.01 | 1125 | 44034 | 670 | 41.17 | 0.21 |
| 2011 | Guangxi | 236000 | 195.34 | 44.82 | 465 | 33132 | 389 | 45.06 | 0.33 |
| 2011 | Guizhou | 176000 | 197.67 | 51.45 | 621 | 24957 | 333 | 39.87 | 0.32 |
| 2011 | Hebei | 187700 | 383.25 | 33.46 | 1247 | 78246 | 598 | 62.82 | 0.46 |
| 2011 | Heilongjiang | 473000 | 81.04 | 25.35 | 911 | 20142 | 646 | 28.28 | 0.54 |
| 2011 | Henan | 167000 | 563.20 | 41.56 | 1220 | 74208 | 560 | 72.21 | 0.35 |
| 2011 | Hubei | 185900 | 308.12 | 29.87 | 608 | 34509 | 425 | 59.43 | 0.41 |
| 2011 | Hunan | 211800 | 310.20 | 37.72 | 782 | 58214 | 526 | 54.29 | 0.41 |
| 2011 | Jiangsu | 102600 | 766.99 | 31.39 | 1283 | 29659 | 497 | 55.38 | 0.35 |
| 2011 | Jiangxi | 167000 | 267.20 | 41.86 | 542 | 38063 | 474 | 44.14 | 0.34 |
| 2011 | Jilin | 187400 | 146.56 | 25.59 | 560 | 18882 | 260 | 39.34 | 0.50 |
| 2011 | Liaoning | 145900 | 299.86 | 27.76 | 831 | 33712 | 492 | 45.10 | 0.29 |
| 2011 | Neimenggu | 1183000 | 20.90 | 27.60 | 488 | 21905 | 446 | 26.70 | 0.52 |
| 2011 | Qinghai | 722300 | 7.79 | 37.41 | 131 | 5608 | 144 | 12.84 | 0.46 |
| 2011 | Shaanxi | 205600 | 181.67 | 30.27 | 871 | 35033 | 384 | 45.97 | 0.41 |
| 2011 | Shandong | 153800 | 623.40 | 34.37 | 1490 | 65954 | 681 | 65.63 | 0.30 |
| 2011 | Shanghai | 6300 | 3655.02 | 23.06 | 308 | 4289 | 101 | 44.02 | 0.57 |
| 2011 | Shanxi | 156300 | 228.67 | 32.75 | 1206 | 38587 | 469 | 56.01 | 0.31 |
| 2011 | Sichuan | 481400 | 167.12 | 38.73 | 1387 | 73646 | 701 | 29.78 | 0.51 |
| 2011 | Tianjin | 11300 | 1149.81 | 22.42 | 296 | 3981 | 95 | 79.40 | 0.40 |
| 2011 | Xinjiang | 1660000 | 13.16 | 36.86 | 820 | 16120 | 464 | 44.96 | 0.47 |
| 2011 | Yunnan | 383300 | 120.05 | 39.57 | 845 | 21800 | 515 | 28.85 | 0.36 |
| 2011 | Zhejiang | 102000 | 533.97 | 29.11 | 731 | 29207 | 372 | 41.24 | 0.29 |
| 2013 | Anhui | 139700 | 428.63 | 39.72 | 938 | 21872 | 1748 | 55.32 | 0.38 |
| 2013 | Beijing | 16800 | 1231.73 | 21.91 | 596 | 8857 | 118 | 68.83 | 0.35 |
| 2013 | Chongqing | 82300 | 357.84 | 41.5 | 531 | 18025 | 350 | 49.77 | 0.43 |
| 2013 | Fujian | 121300 | 308.99 | 33.89 | 541 | 26151 | 1406 | 30.41 | 0.26 |
| 2013 | Gansu | 454400 | 56.72 | 34.75 | 419 | 25514 | 718 | 37.79 | 0.38 |
| 2013 | Guangdong | 180000 | 588.56 | 30.47 | 1222 | 44470 | 1965 | 40.40 | 0.16 |
| 2013 | Guangxi | 236000 | 198.39 | 46.87 | 476 | 32117 | 1288 | 45.69 | 0.27 |
| 2013 | Guizhou | 176000 | 197.96 | 47.33 | 991 | 26657 | 1498 | 39.38 | 0.32 |
| 2013 | Hebei | 187700 | 388.25 | 37.06 | 1268 | 75178 | 1689 | 66.60 | 0.41 |
| 2013 | Heilongjiang | 473000 | 81.06 | 26.52 | 993 | 18883 | 1433 | 28.35 | 0.36 |
| 2013 | Henan | 167000 | 563.23 | 41.59 | 1402 | 67281 | 2472 | 72.78 | 0.30 |
| 2013 | Hubei | 185900 | 310.87 | 33.06 | 711 | 34042 | 779 | 57.61 | 0.38 |
| 2013 | Hunan | 211800 | 313.45 | 42.35 | 922 | 58519 | 2612 | 50.61 | 0.47 |
| 2013 | Jiangsu | 102600 | 771.93 | 32.73 | 1490 | 28815 | 466 | 59.88 | 0.32 |
| 2013 | Jiangxi | 167000 | 269.70 | 42.05 | 548 | 37425 | 823 | 41.62 | 0.33 |
| 2013 | Jilin | 187400 | 146.77 | 24.98 | 576 | 18968 | 293 | 39.46 | 0.42 |
| 2013 | Liaoning | 145900 | 300.82 | 25.5 | 905 | 33521 | 1035 | 41.83 | 0.22 |
| 2013 | Neimenggu | 1183000 | 21.05 | 27.76 | 566 | 21984 | 629 | 26.42 | 0.45 |
| 2013 | Qinghai | 722300 | 7.94 | 38.17 | 145 | 5701 | 170 | 13.70 | 0.54 |
| 2013 | Shaanxi | 205600 | 182.54 | 30.44 | 937 | 34118 | 1962 | 40.94 | 0.35 |
| 2013 | Shandong | 153800 | 629.71 | 36.33 | 1783 | 72108 | 1338 | 67.81 | 0.24 |
| 2013 | Shanghai | 6300 | 3778.46 | 21.18 | 328 | 4439 | 116 | 53.69 | 0.39 |
| 2013 | Shanxi | 156300 | 231.02 | 31.01 | 1219 | 38529 | 462 | 49.71 | 0.27 |
| 2013 | Sichuan | 481400 | 167.76 | 38.97 | 1716 | 75161 | 2969 | 30.59 | 0.58 |
| 2013 | Tianjin | 11300 | 1250.58 | 28.52 | 333 | 4209 | 96 | 82.49 | 0.39 |
| 2013 | Xinjiang | 1660000 | 13.45 | 37.53 | 860 | 17190 | 605 | 48.43 | 0.40 |
| 2013 | Yunnan | 383300 | 121.55 | 37.57 | 997 | 21913 | 1294 | 28.19 | 0.34 |
| 2013 | Zhejiang | 102000 | 536.96 | 26.72 | 843 | 28655 | 396 | 41.41 | 0.22 |
| 2015 | Anhui | 139700 | 435.43 | 39.47 | 1018 | 22030 | 1721 | 49.92 | 0.32 |
| 2015 | Beijing | 16800 | 1280.71 | 23.02 | 631 | 8912 | 113 | 60.99 | 0.38 |
| 2015 | Chongqing | 82300 | 363.48 | 41.61 | 631 | 18986 | 159 | 41.79 | 0.44 |
| 2015 | Fujian | 121300 | 313.77 | 33.66 | 570 | 25876 | 1401 | 27.14 | 0.27 |
| 2015 | Gansu | 454400 | 57.02 | 33.88 | 443 | 25459 | 1774 | 34.72 | 0.33 |
| 2015 | Guangdong | 180000 | 595.78 | 32.88 | 1323 | 45013 | 1831 | 31.95 | 0.19 |
| 2015 | Guangxi | 236000 | 201.44 | 45.84 | 527 | 32216 | 1657 | 36.35 | 0.32 |
| 2015 | Guizhou | 176000 | 199.32 | 45.61 | 1188 | 26175 | 1318 | 34.25 | 0.35 |
| 2015 | Hebei | 187700 | 393.38 | 38.78 | 1543 | 75562 | 1264 | 57.92 | 0.42 |
| 2015 | Heilongjiang | 473000 | 81.04 | 26.55 | 1012 | 18386 | 1301 | 32.21 | 0.45 |
| 2015 | Henan | 167000 | 565.03 | 42.22 | 1521 | 67092 | 2471 | 63.90 | 0.34 |
| 2015 | Hubei | 185900 | 312.86 | 35.56 | 869 | 34563 | 578 | 49.01 | 0.40 |
| 2015 | Hunan | 211800 | 318.09 | 41.19 | 1173 | 58586 | 2778 | 44.34 | 0.41 |
| 2015 | Jiangsu | 102600 | 775.83 | 34.82 | 1581 | 28841 | 1244 | 55.40 | 0.31 |
| 2015 | Jiangxi | 167000 | 271.99 | 42.83 | 568 | 37066 | 812 | 35.65 | 0.33 |
| 2015 | Jilin | 187400 | 146.87 | 28.84 | 616 | 19409 | 432 | 44.71 | 0.40 |
| 2015 | Liaoning | 145900 | 300.96 | 28.91 | 1020 | 33105 | 955 | 46.81 | 0.26 |
| 2015 | Neimenggu | 1183000 | 21.17 | 29.69 | 702 | 22421 | 664 | 29.85 | 0.44 |
| 2015 | Qinghai | 722300 | 8.08 | 34.05 | 181 | 5860 | 178 | 12.39 | 0.47 |
| 2015 | Shaanxi | 205600 | 183.61 | 34.26 | 1014 | 34098 | 1804 | 36.43 | 0.33 |
| 2015 | Shandong | 153800 | 636.50 | 37.47 | 1927 | 73041 | 2086 | 69.71 | 0.29 |
| 2015 | Shanghai | 6300 | 3850.29 | 24.67 | 338 | 4480 | 116 | 49.65 | 0.33 |
| 2015 | Shanxi | 156300 | 233.39 | 30.49 | 1274 | 39196 | 460 | 43.55 | 0.31 |
| 2015 | Sichuan | 481400 | 169.09 | 43.28 | 1942 | 76214 | 1801 | 24.12 | 0.48 |
| 2015 | Tianjin | 11300 | 1342.31 | 28.97 | 402 | 4618 | 151 | 73.06 | 0.46 |
| 2015 | Xinjiang | 1660000 | 13.85 | 38.89 | 914 | 17075 | 802 | 45.79 | 0.36 |
| 2015 | Yunnan | 383300 | 122.98 | 38.27 | 1101 | 21833 | 1183 | 25.98 | 0.34 |
| 2015 | Zhejiang | 102000 | 540.00 | 27.63 | 1049 | 29431 | 483 | 38.55 | 0.25 |
| 2018 | Anhui | 139700 | 447.75 | 47.26 | 1140 | 23076 | 604 | 40.76 | 0.29 |
| 2018 | Beijing | 16800 | 1292.08 | 30.57 | 648 | 9172 | 110 | 40.51 | 0.34 |
| 2018 | Chongqing | 82300 | 373.65 | 44.24 | 800 | 19535 | 153 | 31.47 | 0.15 |
| 2018 | Fujian | 121300 | 322.42 | 38.91 | 641 | 26423 | 450 | 23.36 | 0.15 |
| 2018 | Gansu | 454400 | 57.79 | 38.65 | 626 | 25785 | 1394 | 31.41 | 0.24 |
| 2018 | Guangdong | 180000 | 620.50 | 32.59 | 1553 | 48684 | 1058 | 28.25 | 0.16 |
| 2018 | Guangxi | 236000 | 206.99 | 47.06 | 624 | 31826 | 1257 | 30.20 | 0.19 |
| 2018 | Guizhou | 176000 | 203.41 | 45.43 | 1309 | 26374 | 343 | 26.73 | 0.09 |
| 2018 | Hebei | 187700 | 400.61 | 42.37 | 2108 | 82236 | 684 | 42.68 | 0.29 |
| 2018 | Heilongjiang | 473000 | 80.10 | 28.34 | 1105 | 18460 | 736 | 22.38 | 0.31 |
| 2018 | Henan | 167000 | 572.40 | 46.41 | 1825 | 67730 | 1589 | 49.74 | 0.25 |
| 2018 | Hubei | 185900 | 317.48 | 38.99 | 995 | 34912 | 504 | 36.16 | 0.25 |
| 2018 | Hunan | 211800 | 323.90 | 44.05 | 1552 | 53788 | 851 | 32.72 | 0.26 |
| 2018 | Jiangsu | 102600 | 782.58 | 37.7 | 1853 | 30295 | 808 | 45.54 | 0.18 |
| 2018 | Jiangxi | 167000 | 276.77 | 45.69 | 716 | 35020 | 739 | 29.17 | 0.18 |
| 2018 | Jilin | 187400 | 144.98 | 32.69 | 780 | 21371 | 406 | 26.46 | 0.27 |
| 2018 | Liaoning | 145900 | 299.45 | 31.96 | 1369 | 33777 | 672 | 32.50 | 0.21 |
| 2018 | Neimenggu | 1183000 | 21.38 | 32.25 | 818 | 23235 | 488 | 24.12 | 0.36 |
| 2018 | Qinghai | 722300 | 8.28 | 38.76 | 220 | 5994 | 178 | 12.19 | 0.20 |
| 2018 | Shaanxi | 205600 | 186.53 | 36.48 | 1175 | 33412 | 613 | 32.04 | 0.27 |
| 2018 | Shandong | 153800 | 650.57 | 44.13 | 2580 | 77614 | 1081 | 47.67 | 0.23 |
| 2018 | Shanghai | 6300 | 3838.10 | 31.94 | 358 | 4729 | 108 | 34.95 | 0.13 |
| 2018 | Shanxi | 156300 | 236.85 | 32.61 | 1368 | 40198 | 449 | 39.14 | 0.21 |
| 2018 | Sichuan | 481400 | 172.46 | 42.37 | 2344 | 78427 | 697 | 19.94 | 0.22 |
| 2018 | Tianjin | 11300 | 1377.88 | 29.16 | 420 | 5101 | 96 | 52.73 | 0.25 |
| 2018 | Xinjiang | 1660000 | 14.73 | 43.19 | 907 | 16833 | 697 | 46.21 | 0.50 |
| 2018 | Yunnan | 383300 | 125.24 | 37.62 | 1280 | 23108 | 523 | 22.66 | 0.20 |
| 2018 | Zhejiang | 102000 | 554.61 | 32.72 | 1288 | 30883 | 393 | 28.30 | 0.16 |

**Table S3** The coefficient and p-value of the impact of average PM_2.5_ content on PCMC in the GTWR

| Province | 2011 | | 2013 | | 2015 | | 2018 | |
| --- | --- | --- | --- | --- | --- | --- | --- | --- |
|  | C* | P* | C | P | C | P | C | P |
| Anhui | 0.1302397889 | 0.0622128311 | 0.1882396345 | 0.0020290395 | 0.2633854523 | 0.0000393525 | 0.3416355843 | 0.0000757966 |
| Beijing | 0.0447305810 | 0.3016014140 | 0.1242817968 | 0.0339602285 | 0.2110158503 | 0.0008667495 | 0.2898368234 | 0.0006038165 |
| Chongqing | 0.0323566695 | 0.3389164238 | 0.0829249928 | 0.0779916559 | 0.1810351588 | 0.0014769698 | 0.2909680522 | 0.0002299637 |
| Fujian | 0.2211749287 | 0.0096679252 | 0.2660349127 | 0.0000957892 | 0.3308098770 | 0.0000062649 | 0.4029933612 | 0.0000475811 |
| Gansu | -0.0551294885 | 0.2382111172 | -0.0097219533 | 0.4336569578 | 0.0940187007 | 0.0590288952 | 0.2261076799 | 0.0030828469 |
| Guangdong | 0.2161979855 | 0.0123504712 | 0.2532224464 | 0.0002013564 | 0.3270695682 | 0.0000105124 | 0.4113159831 | 0.0000444210 |
| Guangxi | 0.1525486307 | 0.0461637147 | 0.2004838082 | 0.0015413035 | 0.2924016977 | 0.0000333945 | 0.3881110849 | 0.0000471954 |
| Guizhou | 0.0699568580 | 0.1956152907 | 0.1208044899 | 0.0235524827 | 0.2189292983 | 0.0003346718 | 0.3245501099 | 0.0001129317 |
| Hebei | 0.0495119734 | 0.2706673221 | 0.1191783669 | 0.0320936848 | 0.2048177567 | 0.0006743943 | 0.2902591363 | 0.0002650680 |
| Heilongjiang | 0.0161009370 | 0.4432842167 | 0.1384464582 | 0.0462701815 | 0.2444895333 | 0.0023890644 | 0.3013357490 | 0.0116495999 |
| Henan | 0.0706084591 | 0.1851984687 | 0.1314088832 | 0.0174973668 | 0.2146242481 | 0.0003036185 | 0.3023748683 | 0.0001227869 |
| Hubei | 0.1217521672 | 0.0670845820 | 0.1744770238 | 0.0029912242 | 0.2520299897 | 0.0000543196 | 0.3359562302 | 0.0000567467 |
| Hunan | 0.1382763500 | 0.0475032692 | 0.1861359589 | 0.0018144600 | 0.2642452613 | 0.0000371976 | 0.3501067267 | 0.0000478562 |
| Jiangsu | 0.1358014139 | 0.0582093854 | 0.1969538422 | 0.0015698439 | 0.2713801149 | 0.0000326265 | 0.3465559764 | 0.0000855724 |
| Jiangxi | 0.1613359766 | 0.0304699357 | 0.2098741039 | 0.0007271934 | 0.2823850585 | 0.0000185403 | 0.3620515041 | 0.0000491218 |
| Jilin | 0.0258297883 | 0.4066531973 | 0.1388738668 | 0.0360827431 | 0.2380581236 | 0.0013731709 | 0.2982564081 | 0.0081209381 |
| Liaoning | 0.0399901201 | 0.3503294097 | 0.1415754742 | 0.0283848344 | 0.2340279402 | 0.0009236219 | 0.2988039194 | 0.0044403346 |
| Neimenggu | 0.0118656743 | 0.4391881499 | 0.0813545221 | 0.0941565868 | 0.1719520759 | 0.0024805608 | 0.2660934904 | 0.0004007356 |
| Qinghai | -0.0809491704 | 0.1634235211 | -0.0406812434 | 0.2558426589 | 0.0670245442 | 0.1487888400 | 0.2113160583 | 0.0087696842 |
| Shaanxi | 0.0215935277 | 0.3856599065 | 0.0761986650 | 0.0935876032 | 0.1683273294 | 0.0021271075 | 0.2724150227 | 0.0002519738 |
| Shandong | 0.0758638964 | 0.1839742159 | 0.1471959283 | 0.0133760193 | 0.2294484082 | 0.0002628527 | 0.3083765312 | 0.0002265674 |
| Shanghai | 0.1576087779 | 0.0395143328 | 0.2212096631 | 0.0006382621 | 0.2935481724 | 0.0000174712 | 0.3639301214 | 0.0000884193 |
| Shanxi | 0.0363117434 | 0.3202738954 | 0.1016777441 | 0.0501005113 | 0.1892013936 | 0.0010627788 | 0.2807877892 | 0.0002334094 |
| Sichuan | -0.0162959031 | 0.4188386619 | 0.0315496829 | 0.2953060201 | 0.1368660725 | 0.0122960885 | 0.2607442087 | 0.0009361524 |
| Tianjin | 0.0534382853 | 0.2673736986 | 0.1317879720 | 0.0263149749 | 0.2173539758 | 0.0006285995 | 0.2953914740 | 0.0004965776 |
| Xinjiang | -0.2443439508 | 0.1311532063 | -0.1772395282 | 0.1373027556 | 0.0293761781 | 0.4394030656 | 0.2303739914 | 0.1546970440 |
| Yunnan | 0.0238089848 | 0.3930946830 | 0.0776551575 | 0.1084963506 | 0.1910760012 | 0.0020686740 | 0.3107217637 | 0.0004317745 |
| Zhejiang | 0.1662586134 | 0.0310470728 | 0.2245499338 | 0.0004946898 | 0.2953264188 | 0.0000142406 | 0.3673838725 | 0.0000671458 |
| ***NOTE***：“C” represents regression coefficient and “P” represents significance. | | | | | | | | |


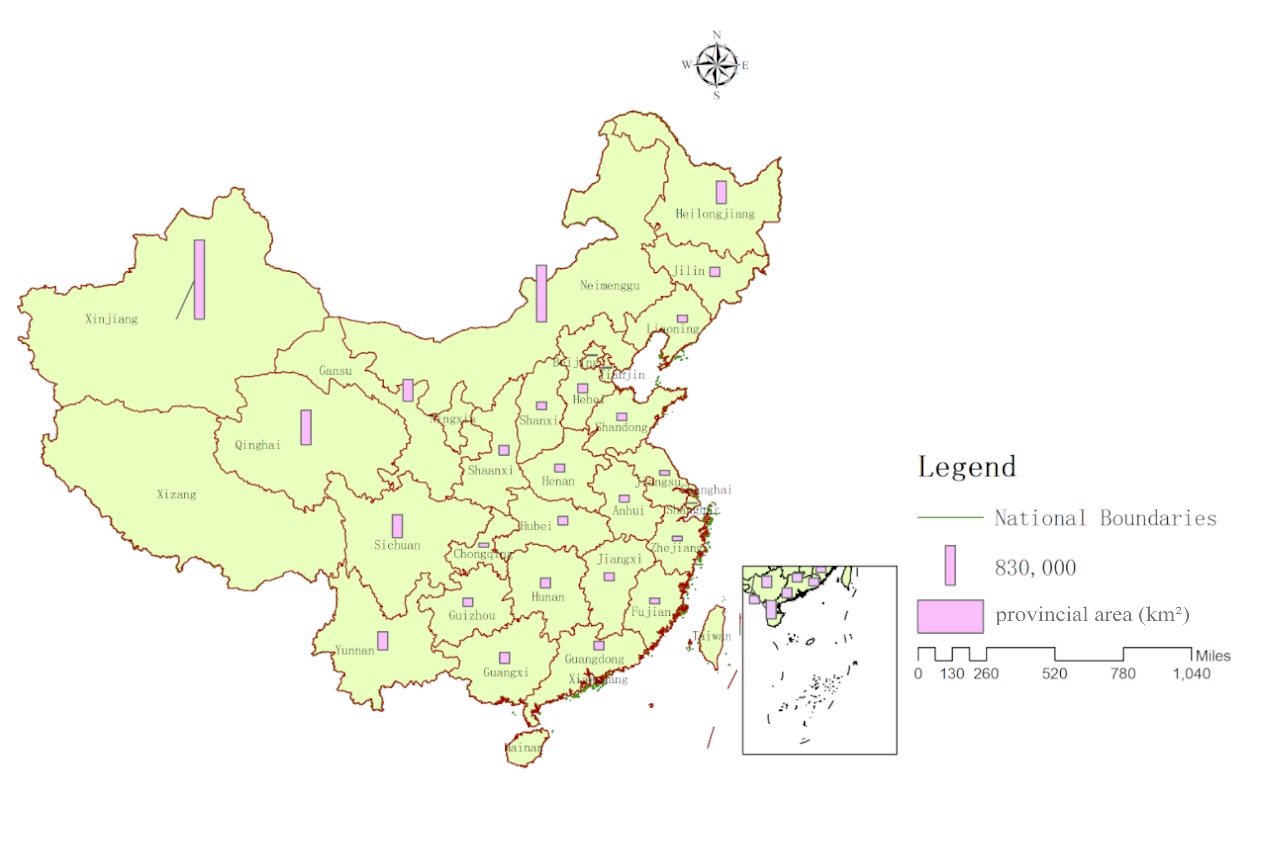


**Figure S1** Spatial representation of the area of each province


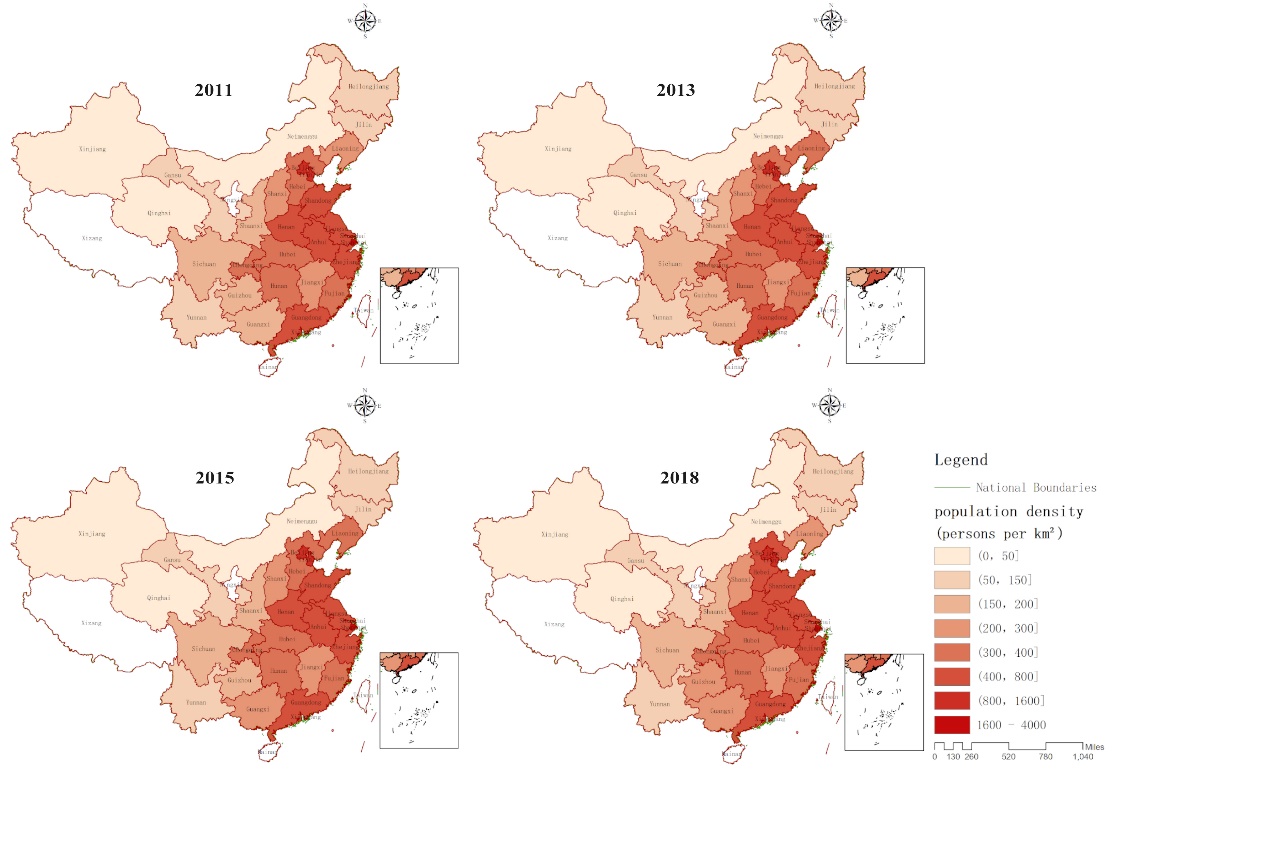


**Figure S2** Spatial representation of population density in each province


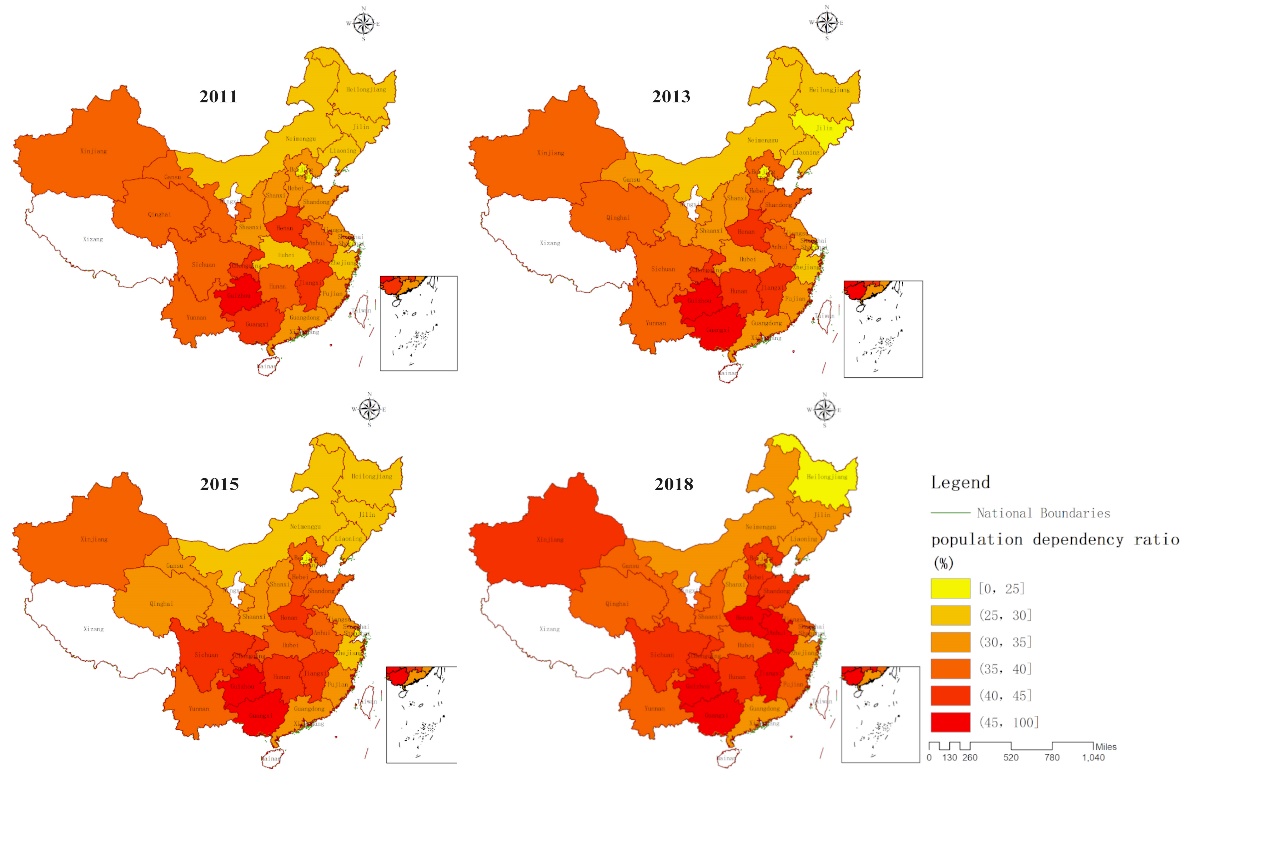


**Figure S3** Spatial representation of population dependency ratio in each province


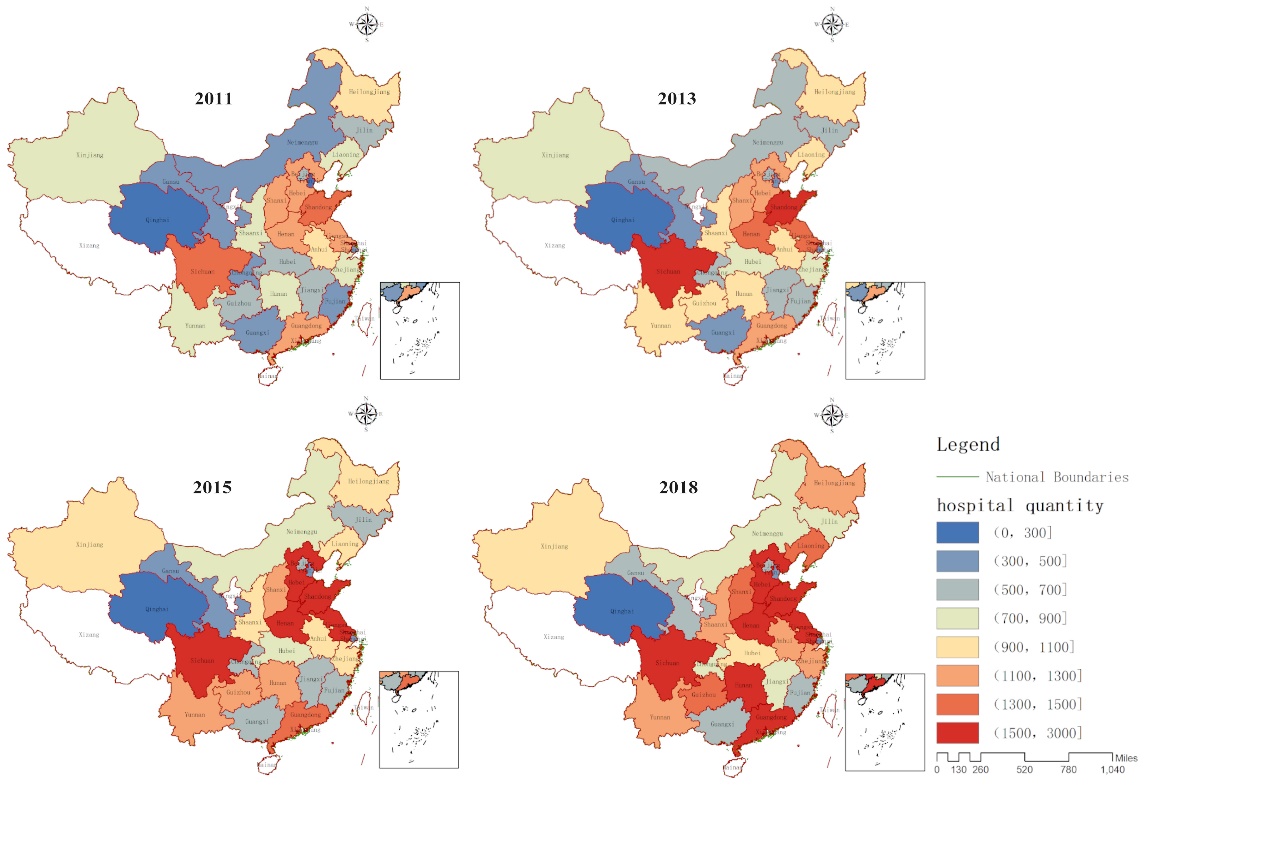


**Figure S4** Spatial representation of hospital quantity in each province


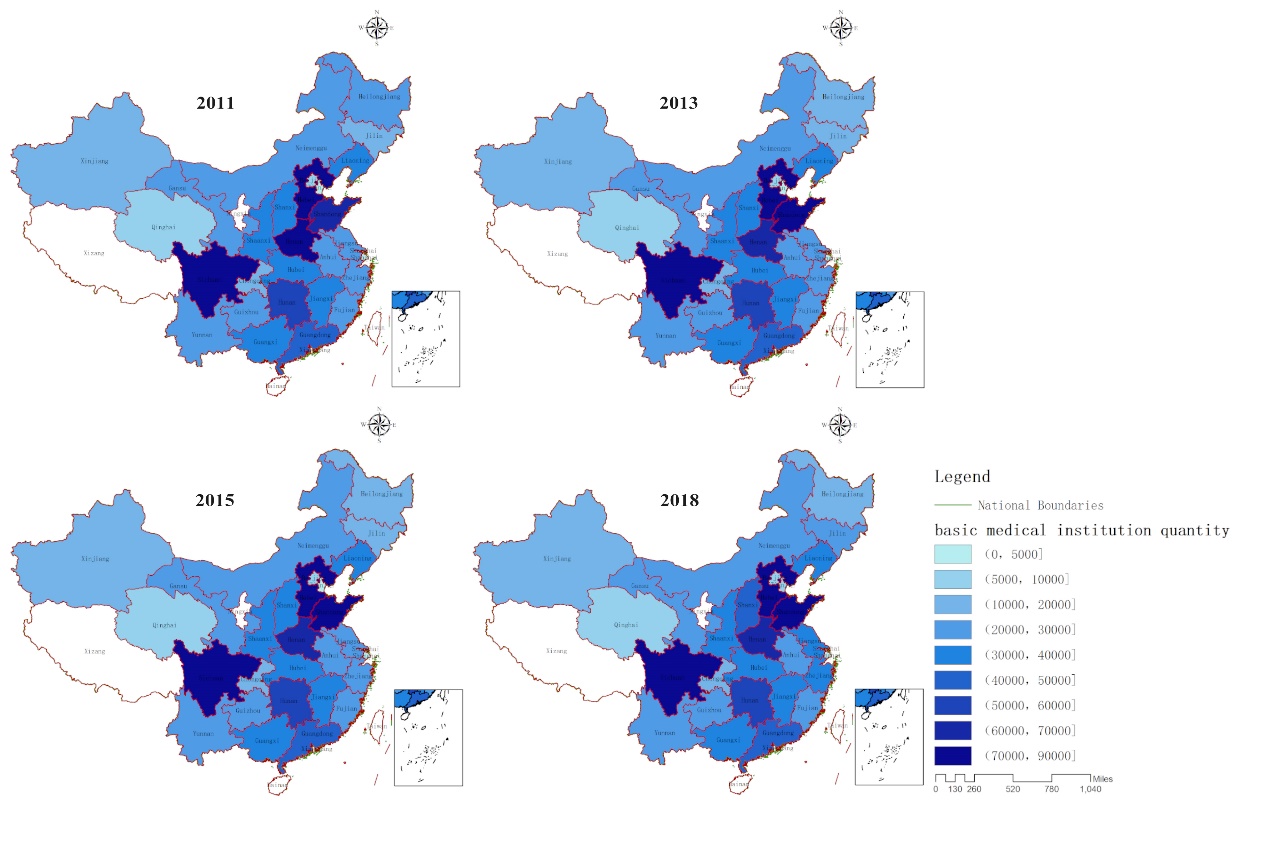


**Figure S5** Spatial representation of basic medical institution quantity in each province


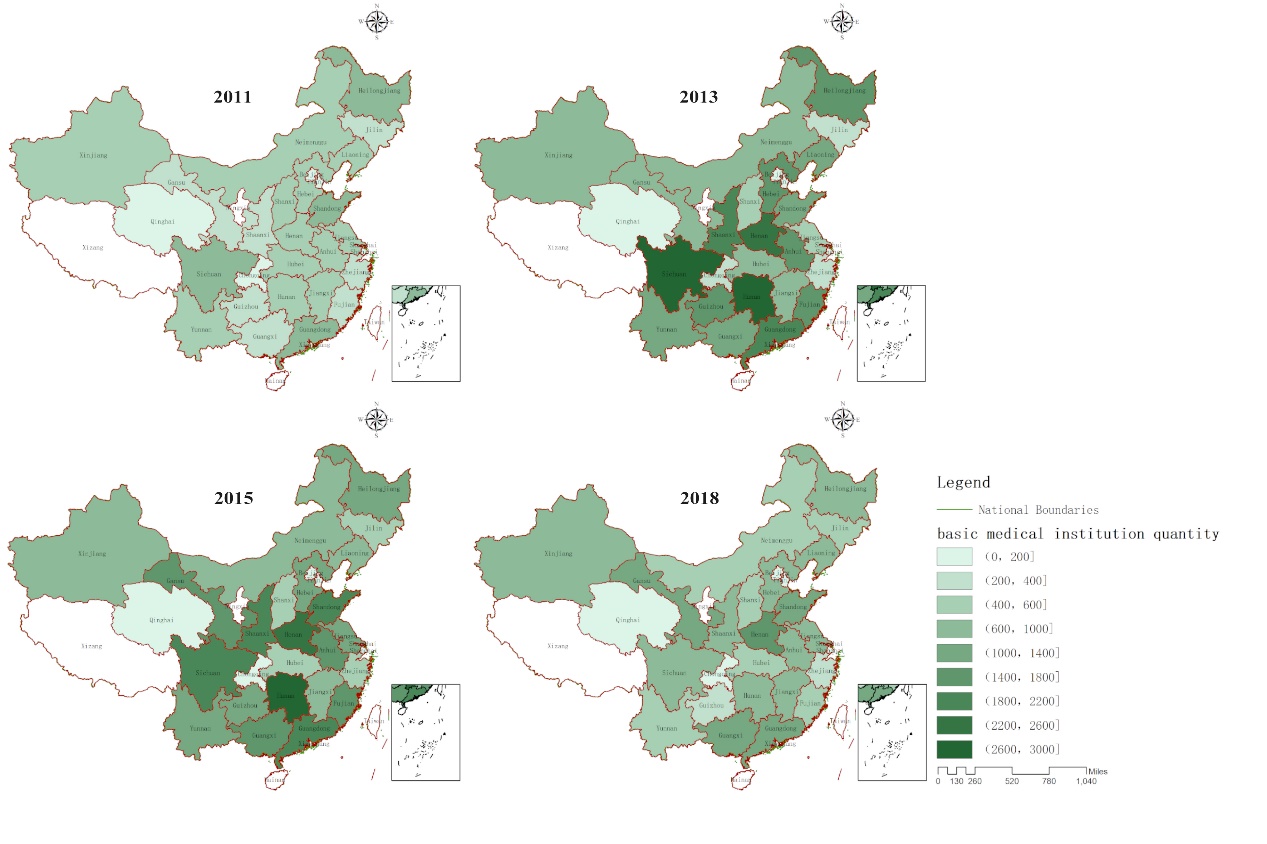


**Figure S6** Spatial representation of specialized public health institution quantity in each province


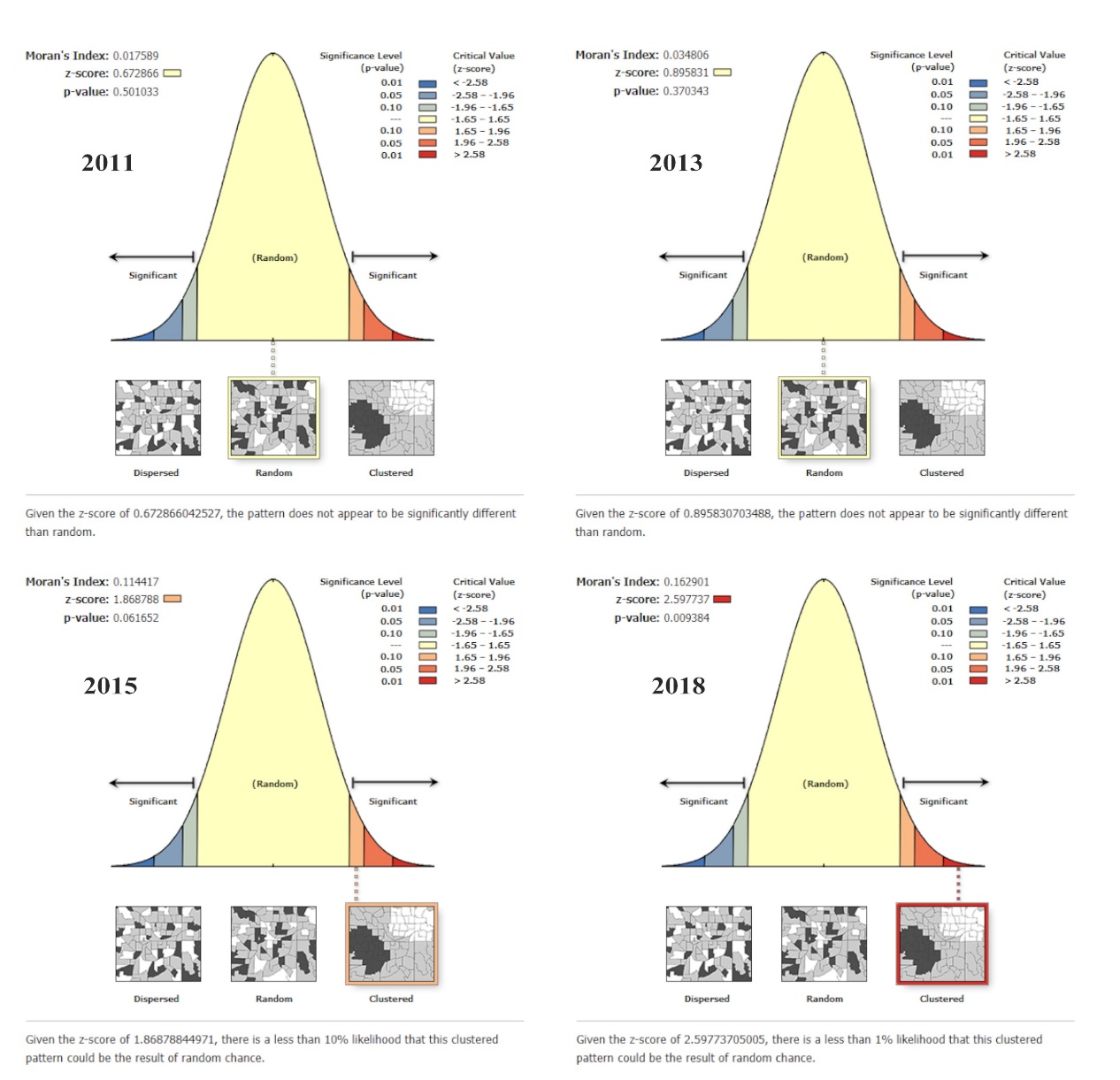


**Figure S7** Global Moran's I result for PCMC in different years


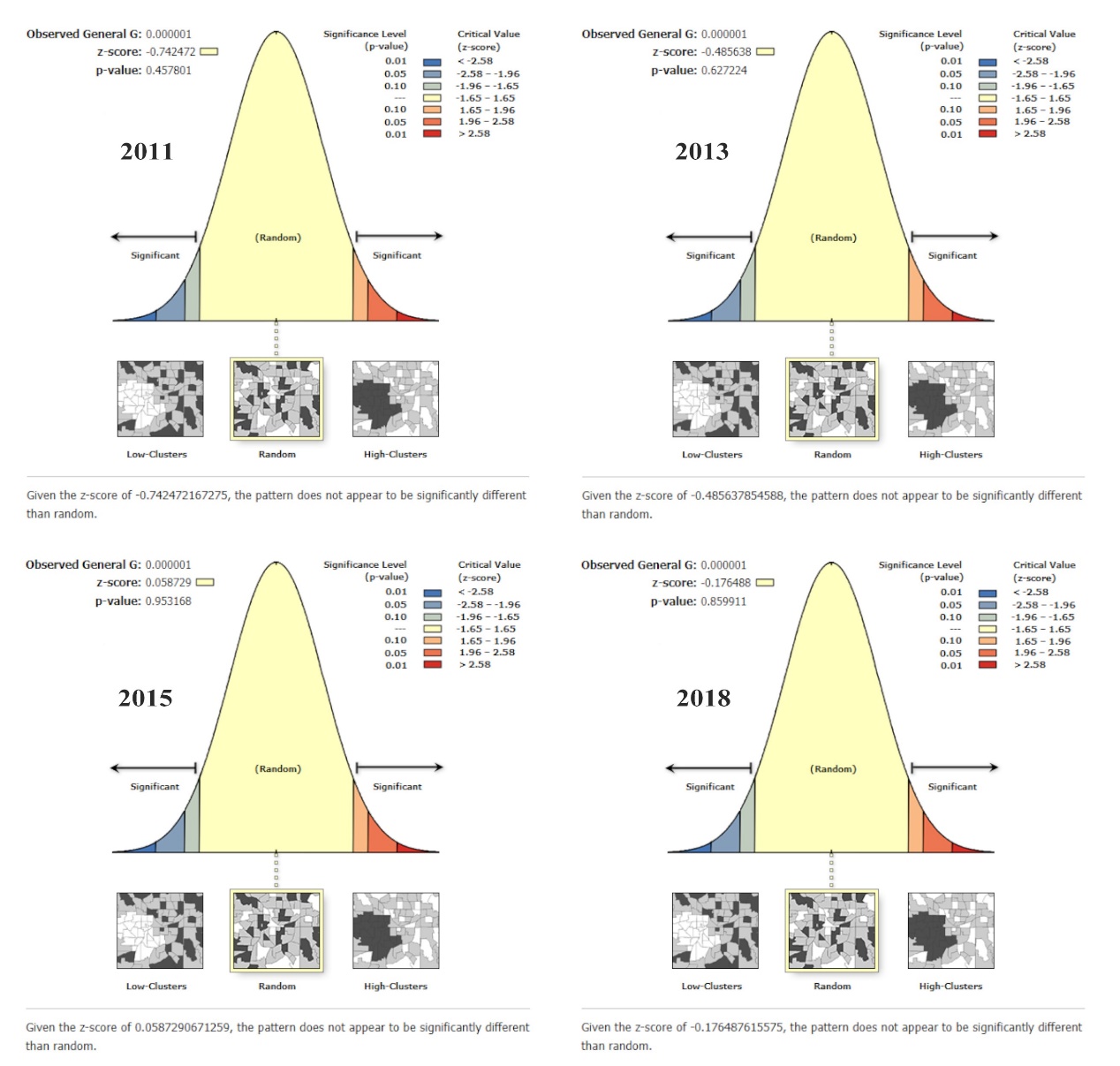


**Figure S8** Getis-Ord General G result for PCMC in different years
